# Fluorination of some highly functionalized cycloalkanes: chemoselectivity and substrate dependence

**DOI:** 10.3762/bjoc.13.233

**Published:** 2017-11-06

**Authors:** Attila Márió Remete, Melinda Nonn, Santos Fustero, Matti Haukka, Ferenc Fülöp, Loránd Kiss

**Affiliations:** 1 Institute of Pharmaceutical Chemistry, University of Szeged, H-6720 Szeged, Eötvös u. 6, Hungary; 2 MTA-SZTE Stereochemistry Research Group, Hungarian Academy of Sciences, H-6720 Szeged, Eötvös u. 6, Hungary; 3 Departamento de Química Orgánica, Facultad de Farmàcia, Universidad de Valencia, Av. Vicente Andrés Estellés, s/n 46100 Valencia, Spain; 4 Department of Chemistry, University of Jyväskylä, FIN-40014, Jyväskylä, Finland

**Keywords:** amino acids, chemoselectivity, fluorine, functionalization, stereoisomers

## Abstract

A study exploring the chemical behavior of some dihydroxylated β-amino ester stereo- and regioisomers, derived from unsaturated cyclic β-amino acids is described. The nucleophilic fluorinations involving hydroxy–fluorine exchange of some highly functionalized alicyclic diol derivatives have been carried out in view of selective fluorination, investigating substrate dependence, neighboring group assistance and chemodifferentiation.

## Introduction

Fluorinated molecules exert an ever-increasing impact in medicinal chemistry thanks to their valuable biological properties. Numerous drugs introduced to the market nowadays contain fluorine, and their number is expected to continuously increase in years to come [1–2]. Therefore, there is a high demand in synthetic organic and medicinal chemistry for both, the synthesis of novel fluorinated biomolecules and the development of selective and controlled efficient fluorination procedures. This high interest is clearly demonstrated by the number of various published papers on the field, and related recent comprehensive reviews [3–12]. The nucleophilic fluorination with commercially available organic fluorinating agents (e.g., DAST, Deoxofluor, Fluolead, XtalFluor-E or XtalFluor-M) is a widely used approach for the introduction of a fluorine atom into an organic molecule. The most common approach is the exchange of a hydroxy group for fluorine, taking place, in general, by inversion [13–18]. Although fluorination based on hydroxy–fluorine interconversion seems to be an eloquent, simple and efficient procedure for the creation of a certain fluorinated organic molecule, regio- and stereoselectivity, stereocontrol and substrate influence remain a challenge in the case of highly functionalized frameworks.

## Results and Discussion

Our aim in this study was to explore the chemical behavior of some alicyclic, highly functionalized vicinal diol derivatives (accordingly possessing two fluorine precursor moieties in their structures) under fluorination protocols. During the past two decades multifunctionalized amino acids and their analogous derivatives have gained considerable interest in pharmaceutical chemistry as bioactive compounds. Some derivatives (e.g., peramivir, oseltamivir or laninamivir) are known as antiviral drugs [19–22], while other cyclopentane β-amino acids (e.g., icofungipen, cispentacin) are relevant antifungal agents [19]. Therefore we have selected some five and six-membered alicyclic dihydroxylated β-amino ester stereo- and regioisomers as model compounds [23–26], derived from cyclopentene or cyclohexene β-amino acids. These were used in order to evaluate their behavior in fluorination in view of selectivity and to explore substrate dependency and chemodifferentiation. Based on the different stereochemical structures of the selected model diols as well as the nature of the *N*-protecting group used, we expected differences in their chemical behavior under fluorination conditions. In a former investigation on oxirane opening reactions of various epoxycycloalkane β-aminocarboxylates [27], a high substrate dependence and directing effect of the functional groups has been observed. These results led us to perform similar investigations with the above mentioned dihydroxylated cyclic β-amino acid esters.

**Scheme 1:**
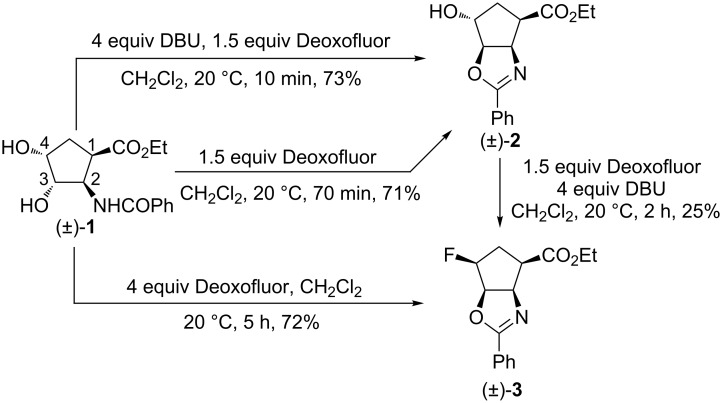
Fluorination of diol derivative (±)-**1**.

Our study started with the fluorination of racemic dihydroxylated cyclopentane *cis*- or *trans*-β-amino acid esters (±)-**1** and (±)-**4** [23–26]. The preliminary investigations were performed with the commercially available fluorinating agents mentioned above in various solvents (e.g., PhMe, THF, and CH_2_Cl_2_) at different temperatures. Hereby Deoxofluor in CH_2_Cl_2_ with or without adding DBU was found to be the most suitable reagent. The diol derivatives, obviously, are expected to deliver the corresponding difluorinated products. In spite of this anticipation, the treatment of dihydroxylated amino acid ester (±)-**1** with 1.5 equiv of Deoxofluor after 70 min afforded through intramolecular cyclization a compound which was identified on the basis of 2D NMR analysis as oxazoline derivative (±)-**2** as the sole product in 71% yield. When the same reaction was carried out in the presence of 4 equiv DBU as the base [28] the reaction time could be decreased to 10 min with a slightly increased yield of the product (Scheme 1). This observation led us to conclude that out of the two hydroxy groups only that attached to C-3 takes part in the reaction and is substituted via the amide O-atom. Increasing the amount of Deoxofluor to 4 equiv resulted in the exclusive formation of the fluorine-containing oxazoline derivative (±)-**3**. Note that the addition of DBU did not have a significant effect on this reaction. When isolated hydroxyoxazoline (±)-**2** is subjected to the fluorination protocol compound (±)-**3** was obtained in a modest yield (15%) that could be slightly increased to 25% by the addition of DBU to the reaction mixture (Scheme 1).

**Scheme 2:**
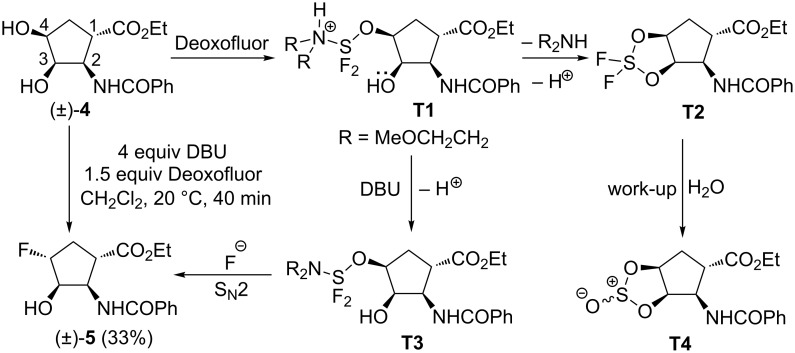
Fluorination of diol derivative (±)-**4**.

**Figure 1:**
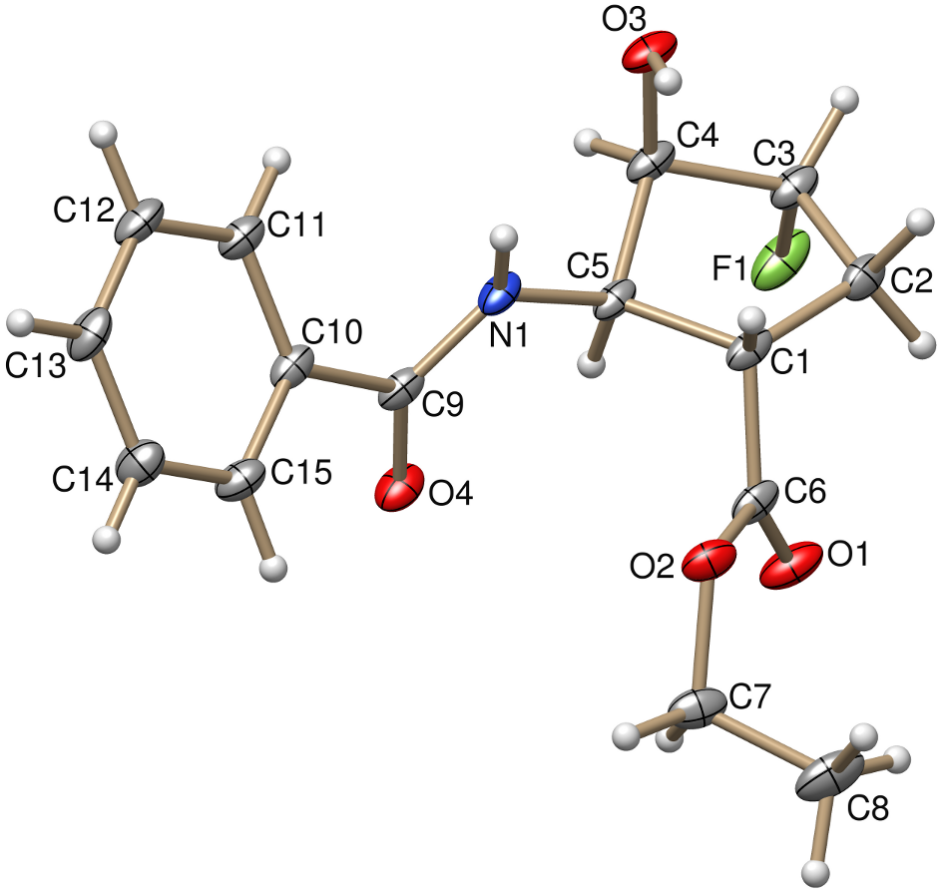
X-ray structure of fluorohydrine derivative (±)-**5**.

In contrast to (±)-**1**, its stereoisomer (±)-**4**, afforded in the reaction with 1.5 equiv of Deoxofluor in the presence of 4 equiv of DBU, according to 2D NMR and X-ray data fluorohydrine derivative (±)-**5** through chemodifferentiation in a moderate yield (Scheme 2, Figure 1).

This result indicated that in the case of compound (±)-**4**, the C-4 hydroxy group is transformed into a leaving group on interaction with Deoxofluor, followed by a fluoride attack to give (±)-**5**. Of note, the role of DBU was found to be crucial in this reaction, since without the addition of the base the formation of sulfite derivative **T4** was detected (Scheme 2).

The vicinal fluorohydrine moiety is a relevant unit in a series of valuable biomolecules such as amino acids, heterocyclic natural products and nucleosides [29–36]. Therefore, the fluorinated β-amino acid derivative (±)-**5**, obtained through transformation of (±)-**4** with chemodiscrimination of the alcoholic groups, might represent a promising bioactive framework.

**Scheme 3:**
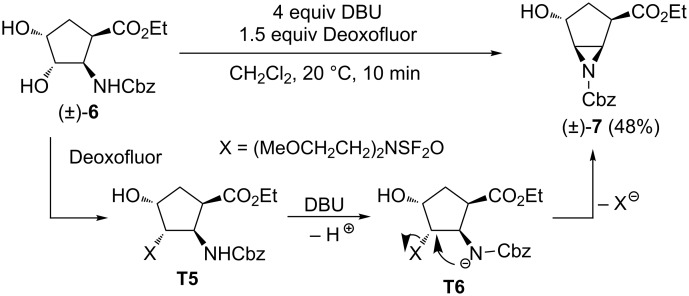
Fluorination of diol derivative (±)-**6**.

As the fluorination of the five-membered diol stereoisomers (±)-**1** and (±)-**4** proved to be highly substrate dependent and on the basis of our earlier findings on substrate determinant fluorinations [37], we next investigated the effect of the nature of the *N*-protecting group on this reaction. The treatment of *N*-Cbz-protected dihydroxylated cyclopentane β-amino ester (±)-**6** with Deoxofluor under various conditions provided an unidentifiable mixture of products. However, the reaction of diol (±)-**6** with 1.5 equiv of Deoxofluor in the presence of DBU as the base furnished aziridine derivative (±)-**7** within 10 min through intramolecular cyclization via the carbamate N-atom (Scheme 3). Again, only the selective transformation of the C-3 hydroxy group with the fluorinating agent took place, without the involvement of the C-4 OH group. Unfortunately, all further attempted fluorinations of hydroxylated aziridine (±)-**7** under various conditions using for example XtalFluor, Et_3_N·HF, and pyridine·HF and applying an efficient approach earlier developed by our group, proved to be unsuccessful ([18] and references cited therein).

**Scheme 4:**
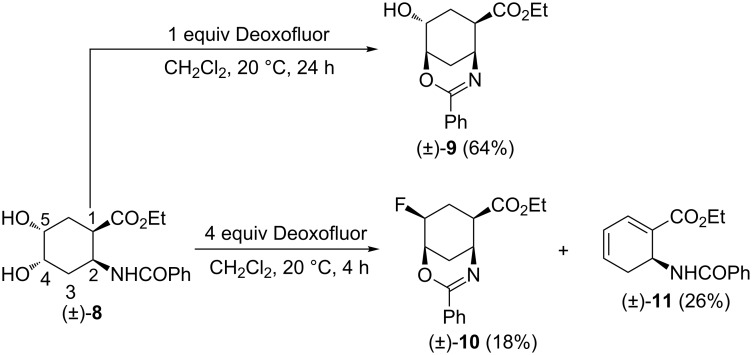
Fluorination of cyclohexane-derived diol (±)-**8**.

Due to the biological relevance of six-membered β-amino acid derivatives (e.g., tilidin, oryzoxymicin, BAYY9379) [19], we continued our synthetic investigations by selecting some cyclohexane β-amino acid esters as model compounds. Thus, diol (±)-**8** [23–26] derived from dihydroxylation of *cis*-2-aminocyclohex-4-ene carboxylic acid was treated with 1 equiv of Deoxofluor in CH_2_Cl_2_. Again, through chemodiscrimination of the alcoholic groups in positions 4 and 5, oxazine derivative (±)-**9** was formed as the single product through intramolecular cyclization involving the nucleophilic amide O-atom (Scheme 4).

**Scheme 5:**
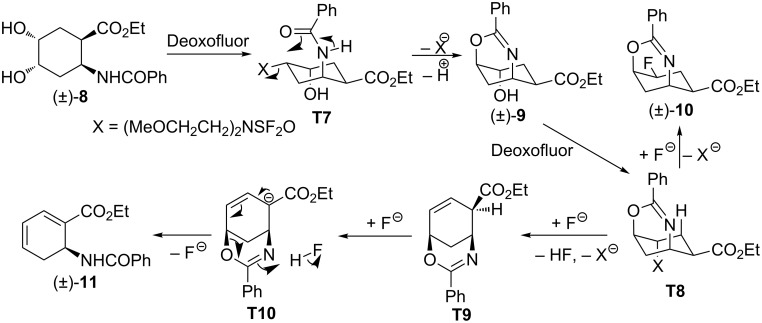
Proposed route for the formation of compounds (±)-**10** and (±)-**11**.

Upon treatment of diol (±)-**8** with excess Deoxofluor (4 equiv), in addition to the expected fluorinated oxazine (±)-**10**, a highly unsaturated amino ester (±)-**11** was formed as the major product. The two compounds were separated by column chromatography and product (±)-**11** was identified to be a cyclohexadiene amino acid ester. A relatively simple way of its formation is depicted in Scheme 5. Specifically, after transformation of the alcoholic group at C-4 in (±)-**8** into a leaving group, an intramolecular cyclization takes place to afford oxazine (±)-**9**. Subsequently, this oxazine, in the presence of an excess Deoxofluor gives fluorinated derivative (±)-**10**. Finally, in the presence of fluoride as base, deprotonation (**T9**), followed by oxazine-ring opening through olefinic-bond migration (**T10**) then gives the highly conjugated amino acid ester (±)-**11** (Scheme 5).

Noteworthy, the presence of DBU in the experiment of (±)-**8** with Deoxofluor did not have a significant effect on the ratio of the products. However, in the presence of the base the yields of (±)-**10** and (±)-**11** decreased to 10% and 17%, respectively.

**Scheme 6:**
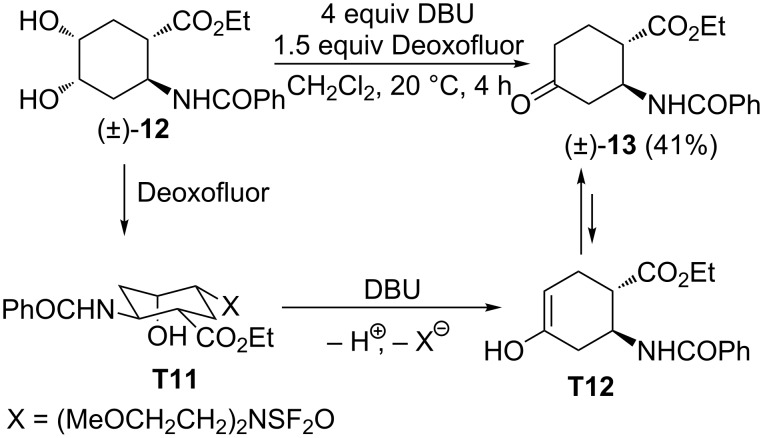
Fluorination of diol derivative (±)-**12**.

Unfortunately and surprisingly, diol (±)-**12** (derived from *trans*-2-aminocyclohex-4-ene carboxylic acid) [23–26] a stereoisomer of (±)-**8**, in the reaction with either 1 equiv or excess of Deoxofluor did not give any identifiable product. However, according to earlier observations on the effect of DBU the reaction in the presence of this base afforded keto ester (±)-**13** in 41% yield. In contrast to the stereoisomer (±)-**8**, reaction of diol (±)-**12** with Deoxofluor took place at the C-5 hydroxy group leading to intermediate **T11**. The latter, in the presence of DBU, gave enol **T12** which immediately converted to keto amino ester (±)-**13** (Scheme 6).

**Scheme 7:**
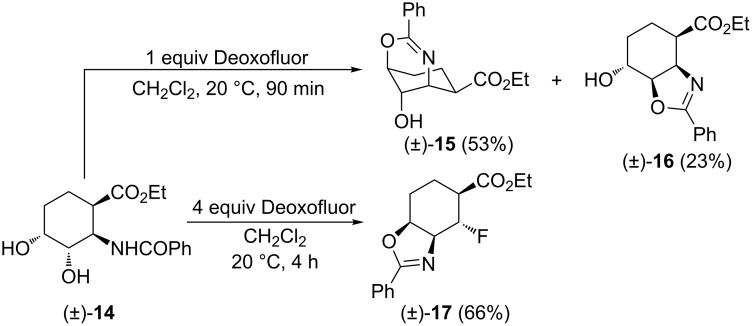
Fluorination of diol derivative (±)-**14**.

Subsequently, we investigated the behavior of other six-membered diol isomers in this reaction. The diol derivative (±)-**14** (derived from *cis*-2-aminocyclohex-3-ene carboxylic acid), underwent intramolecular cyclization upon treatment with 1 equiv of Deoxofluor providing isomers (±)-**15** and (±)-**16** in nearly 2:1 ratio that could be separated and isolated by chromatography. This experimental observation indicates that in the case of diol (±)-**14** both alcoholic groups are transformed into the corresponding good leaving groups upon treatment with Deoxofluor. When (±)-**14** was treated with an excess of Deoxofluor, somewhat surprisingly, the fluorinated isoxazoline derivative (±)-**17** was formed with the fluorine attached to the β-position relative to the ester function, whereas the N-atom is located in the γ-position through a β to γ flip (Scheme 7).

**Scheme 8:**
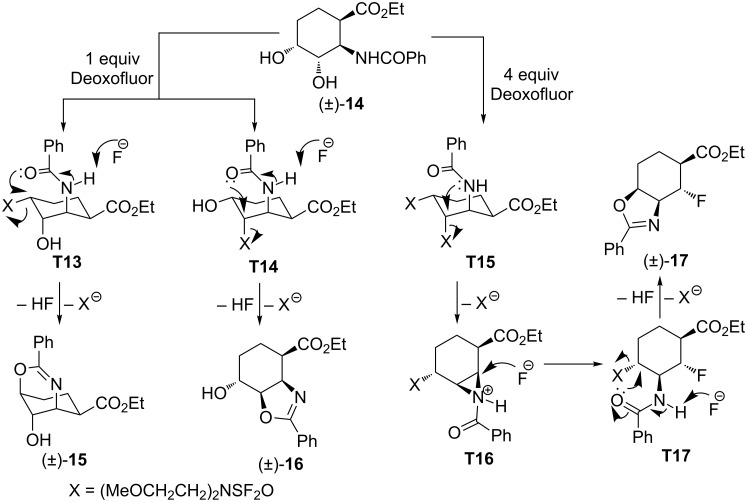
Proposed route for the formation of compounds (±)-**15**, (±)-**16** and (±)-**17**.

The formation of isoxazolines (±)-**15** and (±)-**16** in the presence of 1 equiv of Deoxofluor involves rather simple reaction steps as only one of the hydroxy groups is transformed into a leaving group in each case (**T13** and **T14**, respectively). In contrast, when using the fluorinating reagent in excess, both hydroxy groups are converted to leaving groups to afford intermediate **T15**. The latter affords aziridinium ion **T16** through intramolecular reaction with the amide nitrogen. The subsequent aziridine-ring opening by reaction with fluoride results in amide intermediate **T17** which undergoes an intramolecular cyclization to give (±)-**17** (Scheme 8). A similar aziridinium opening reaction with fluoride can be found in [38–39].

**Scheme 9:**
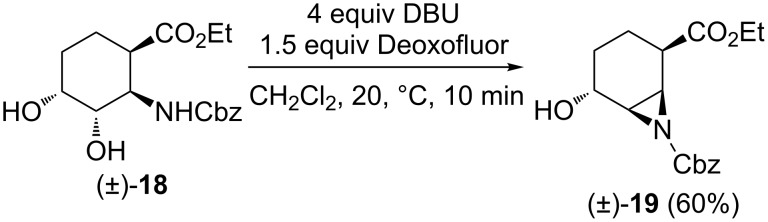
Fluorination of *N*-Cbz-protected diol derivative (±)-**18**.

Interestingly, when changing the *N*-protecting group from benzoyl in (±)-**14** to Cbz ((±)-**18**), the fluorination with Deoxofluor provided, analogously to the five-membered compound (±)-**6** (Scheme 3), aziridine (±)-**19** in 60% yield (Scheme 9).

**Scheme 10:**
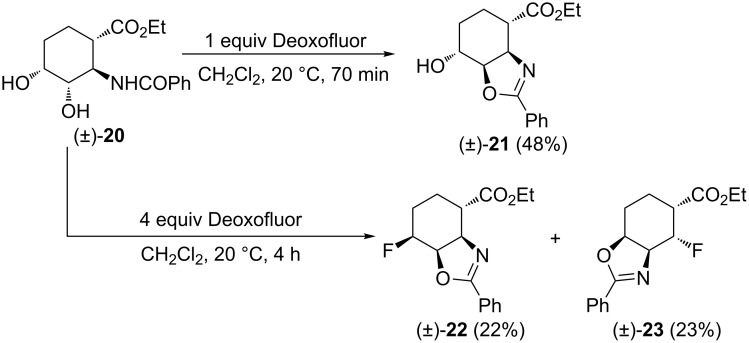
Fluorination of diol derivative (±)-**20**.

Diol (±)-**20** (derived from *trans*-2-aminocyclohex-3-ene carboxylic acid), a stereoisomer of (±)-**14**, furnished selectively one single cyclized product in the presence of 1 equiv of Deoxofluor, namely oxazoline derivative (±)-**21** (Scheme 10). This is in high contrast to the transformation of (±)-**14** (Scheme 7). Interestingly, when (±)-**20** is treated with Deoxofluor in excess, again, differently from its all-*cis* counterpart (±)-**14**, two fluorinated oxazolines in nearly 1:1 ratio are obtained: (±)-**22** as the expected product, with the ring N-atom in the β-position to the ester group and compound (±)-**23**, having the N-atom in the γ-position relative to the carboxylate. The latter being formed by *N*-migration, through an aziridinium ion opening reaction with fluoride.

**Scheme 11:**
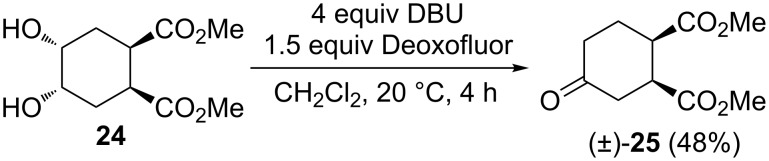
Fluorination of meso diol derivative **24**.

Finally, the fluorination protocol was applied to commercially available *meso*-compound **24** having a cyclohexane framework. However, the treatment of **24** with various amounts of Deoxofluor gave only unidentifiable products whereas the reaction in the presence of DBU, afforded ketoester (±)-**25** (see also Scheme 6) in 48% yield (Scheme 11).

## Conclusion

In conclusion, the chemical behavior of the alcoholic functions of some vicinal diol derivatives towards fluorination has been investigated under various conditions. The fluorination reactions of the highly functionalized cycloalkanes were found to be highly substrate dependent, taking place with chemodiscrimination of the two hydroxy groups. The stereochemical arrangement of the functional groups deeply influenced the selectivity of the transformations. Further investigations of other substrates in view of selectivity and substrate-directing effects are currently being studied in our research group.

## Experimental

### General information

Chemicals were purchased from Sigma–Aldrich and the solvents were used as received. Melting points were determined with a Kofler apparatus. Elemental analyses were carried out with a Perkin–Elmer CHNS-2400 Ser II elemental analyzer. Silica gel 60 F254 was purchased from Merck. The NMR spectra were acquired at room temperature on a Bruker Avance 400 spectrometer with 11.75 T magnetic field strength (^1^H frequency: 400.13 MHz, ^19^F frequency: 376.50 MHz, ^13^C frequency: 100.76 MHz, respectively) in CDCl_3_ or DMSO-*d*_6_ solution, using the deuterium signal of the solvent to lock the field. The ^1^H and ^13^C chemical shifts are given relative to TMS and ^19^F chemical shifts are referenced to CFCl_3_ (0.00 ppm).

### General procedures for fluorination

**Method A:** To a solution of dihydroxylated compound (0.50 mmol) in 10 mL anhydrous CH_2_Cl_2_ under an Ar atmosphere, 50% Deoxofluor in toluene was added (amount given in the text) and the reaction mixture was stirred at 20 °C for the time given in the text. The solution was then diluted with CH_2_Cl_2_ (30 mL) and washed with saturated aqueous NaHCO_3_ solution (2 × 20 mL). The organic layer was dried (Na_2_SO_4_) and concentrated. The crude product was purified by column chromatography on silica gel (*n*-hexane/EtOAc or *n*-hexane/acetone).

**Method B:** To a solution of dihydroxylated compound (0.50 mmol) in 15 mL anhydrous CH_2_Cl_2_ under an Ar atmosphere, 4 equiv DBU and 1.5 equiv 50% Deoxofluor in toluene were added and the solution was stirred at 20 °C for the time given in the text. The solution was then diluted with CH_2_Cl_2_ (30 mL) and washed with saturated aqueous NaHCO_3_ solution (2 × 20 mL). The organic layer was dried (Na_2_SO_4_) and concentrated. The crude product was purified by column chromatography on silica gel (*n*-hexane/EtOAc or *n*-hexane/acetone).

## Supporting Information

File 1 Characterization data and copies of NMR spectra.
