# Supplementary material for: Fluorination of some highly functionalized cycloalkanes: chemoselectivity and substrate dependence
Source: Beilstein J Org Chem. 2017 Nov 6;13:2364–71. doi: 10.3762/bjoc.13.233 (PMC5687045; doi:10.3762/bjoc.13.233)
Supplement: File 1 — Characterization data and copies of NMR spectra. [file Beilstein_J_Org_Chem-13-2364-s001.pdf]

Supporting Information

for

Fluorination of some highly functionalized cycloalkanes: chemoselectivity and substrate dependence

Attila Márió Remete[1], Melinda Nonn[2], Santos Fustero[3], Matti Haukka[4], Ferenc Fülöp[1,2]*, and Loránd Kiss[1]*

Address: [1]Institute of Pharmaceutical Chemistry, University of Szeged, H-6720 Szeged, Eötvös u. 6, Hungary, [2]MTA-SZTE Stereochemistry Research Group, Hungarian Academy of Sciences, H-6720 Szeged, Eötvös u. 6, Hungary, [3]Departamento de Química Orgánica, Facultad de Farmàcia, Universidad de Valencia, Av. Vicente Andrés Estellés, s/n 46100 Valencia, Spain and [4]Department of Chemistry, University of Jyväskylä, FIN-40014, Jyväskylä, Finland

Email: Loránd Kiss - kiss.lorand00@gmail.com; Ferenc Fülöp - fulop@pharm.u-szeged.hu

Characterization data and copies of NMR spectra

(1*S,2*R**,3*R**,4*R**)-Ethyl -2-benzamido-4-fluoro-3-hydroxycyclopentane-carboxylate ((±)-5)**

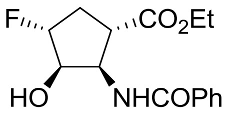

White solid; yield 33%; R_f = 0.40 (*n*-hexane/EtOAc 1:1); Mp 99-103 °C. ^{1}H-NMR (400 MHz, $CDCl_3$, TMS): δ = 1.23 (t, *J* = 7.12 Hz, 3H, CH_3), 2.12-2.31 (m, 1H, H-5), 2.51-2.71 (m, 1H. H-5), 3.08-3.19 (m, 1H, H-1), 4.18 (q, *J* = 7.14 Hz, 2H, OCH_2), 4.41-4.50 (m, 1H, H-3), 4.52-4.62 (m, 1H, H-2), 4.82-5.02 (m, 1H, H-4), 6.78 (d, *J* = 5.88 Hz, 1H, NH), 7.39-7.48 (m, 2H, CH-Ar), 7.48-7.56 (m, 1H, CH-Ar), 7.73-7.82 (m, 2H, CH-Ar). ^{19}F NMR (376 MHz, $CDCl_3$): δ = -175.6. ^{13}C NMR (100 MHz, $CDCl_3$, TMS): δ = 14.5, 32.5 and 32.7 (^{2}J = 23.34 Hz), 45.8, 56.6, 61.7, 75.9 and 76.1 (^{2}J = 27.66 Hz), 95.6 and 97.3 (^{1}J = 179.51 Hz), 127.5, 129.0, 132.2, 134.4, 168.5, 174.1. MS (ESI, pos) m/z=296 [M+1]. Anal. Calcd. for $C_{15}H_{18}FNO_4$: C, 61.01; H, 6.14; N, 4.74. Found: C, 61.30; H, 5.76; N, 4.45.

(3a*R,4*R**,6*R**,6a*R**)-Ethyl -6-hydroxy-2-phenyl-4,5,6,6a-tetrahydro-3a*H*-cyclo-penta[*d*]oxazole-4-carboxylate ((±)-2)**

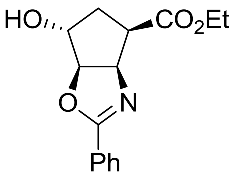

White solid; yield 73%; R_f = 0.47 (*n*-hexane/EtOAc 1:2); Mp 99-105 °C. ^{1}H-NMR (400 MHz, $CDCl_3$, TMS): δ = 1.33 (t, *J* = 7.12 Hz, 3H, CH_3), 1.83-2.06 (m, 2H, H-5), 3.41-3.52 (m, 1H, H-4), 4.18-4.33 (m, 2H, OCH_2), 4.38-4.45 (m, 1H, H-6), 4.80-4.87 (m, 1H, H-6a), 5.03-5.10 (m, 1H, H-3a), 7.31-7.41 (m, 2H, CH-Ar), 7.42-7.50 (m, 1H, CH-

Ar), 7.84-7.92 (m, 2H, CH-Ar). ^{13}C NMR (100 MHz, $CDCl_3$, TMS): δ = 14.7, 33.1, 48.0, 61.1, 72.7, 76.1, 88.6, 128.7, 128.9, 132.0, 164.2, 171.9. MS (ESI, pos) m/z=276 [M+1]. Anal. Calcd. for $C_{15}H_{17}NO_4$: C, 65.14; H, 6.22; N, 5.09. Found: C, 64.89; H, 5.90; N, 4.79.

(3a*S,4*R**,6*S**,6a*R**)-Ethyl -6-fluoro-2-phenyl-4,5,6,6a-tetrahydro-3a*H*-cyclopenta[*d*]oxazole-4-carboxylate ((±)-3)**

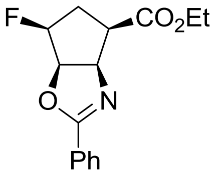

Yellowish white solid; 25%; R_f = 0.65 (*n*-hexane/acetone 2:1); Mp 84-87 °C. ^{1}H-NMR (400 MHz, $CDCl_3$, TMS): δ = 1.34 (t, *J* = 7.14 Hz, 3H, CH_3), 1.88-2.25 (m, 2H, H-6), 3.34-3-46 (m, 1H, H-4), 4.20-4.34 (m, 2H, OCH_2), 4.98-5.18 (m, 3H, H-3a and H-6 and H-6a), 7.33-7.42 (m, 2H, CH-Ar), 7.43-7.51 (m, 1H, CH-Ar), 7.84-7.91 (m, 2H, CH-Ar). ^{19}F NMR (376 MHz, $CDCl_3$): δ = -183.05. ^{13}C NMR (100 MHz, $CDCl_3$, TMS): δ = 14.7, 31.3 and 31.5 (^{2}J = 20.96 Hz), 47.9, 61.2, 72.8, 85.6 and 85.9 (^{2}J = 33.62 Hz), 95.4 and 97.2 (^{1}J = 175.21 Hz), 127.1, 128.7, 129.0, 132.1, 164.0, 171.1. MS (ESI, pos) m/z=278 [M+1]. Anal. Calcd. for $C_{15}H_{16}FNO_3$: C, 64.97; H, 5.82; N, 5.05. Found: C, 64.58; H, 5.51; N, 4.72.

(1*R,2*R**,4*R**,5*R**)-6-Benzyl 2-ethyl 4-hydroxy-6-azabicyclo[3.1.0]hexane-2,6-dicarboxylate ((±)-7)**

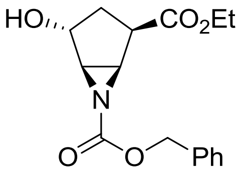

Colorless oil; yield 48%; R_f = 0.33 (*n*-hexane/acetone 5:2). ^{1}H-NMR (400 MHz, D_6-DMSO, TMS) δ = 1.11 (t, *J* = 7.10 Hz, 3H, CH_3), 1.61-1.69 (m, 2H, H-3), 3.00-3.05 (m, 1H, H-5), 3.09-3.17 (m, 1H, H-2), 3.36-3.41 (m, 1H, H-1), 3.96-4.12 (m, 2H, OCH_2), 4.13-4.20 (m, 1H, H-4), 4.99-5.08 (m, 3H, PhC*H₂* and OH), 7.29-7.42 (m, 5H, CH-Ar). ^{13}C NMR (100 MHz, $CDCl_3$, TMS) δ = 14.5, 33.6, 43.4, 44.1, 46.3, 61.4, 68.7, 71.8, 128.6, 128.8, 128.9, 136.0, 162.0, 172.5. MS (ESI, pos) m/z=328 [M+Na]. Anal. Calcd. for $C_{16}H_{19}NO_5$: C, 62.94; H, 6.27; N, 4.59. Found: C, 62.59; H, 5.80; N, 4.21.

(1*S,2*S**)-Ethyl 2-benzamido-4-oxocyclohexanecarboxylate ((±)-13)**

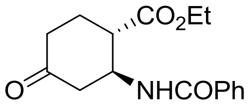

White solid; yield 41%; R_f = 0.47 (*n*-hexane/EtOAc 1:2); Mp 139-144 °C. ^{1}H-NMR (400 MHz, $CDCl_3$, TMS): δ = 1.23 (t, *J* = 7.14 Hz, 3H, CH_3), 2.02-2.28 (m, 2H, H-6), 2.32-2.45 (m, 1H, H-5), 2.49-2.59 (m, 1H, H-5), 2.59-2.69 (m, 1H, H-3), 2.91-3.02 (m, 1H, H-3), 3.09-3.19 (m, 1H, H-1), 4.10-4.24 (m, 2H, OCH_2), 4.51-4.64 (m, 1H, H-2), 6.24 (d, *J*=6.92 Hz, NH), 7.38-7.47 (m, 2H, CH-Ar), 7.47-7.56 (m, 1H, CH-Ar), 7.67-7.77 (m, 2H, CH-Ar). ^{13}C NMR (100 MHz, $CDCl_3$, TMS): δ = 14.5, 25.5, 39.5, 46.2, 46.3, 51.1, 61.6, 127.3, 129.1, 132.2, 134.5, 167.3, 173.1, 207.8. MS (ESI, pos)

m/z=290 [M+1]. Anal. Calcd. for $C_{16}H_{19}NO_4$: C, 66.42; H, 6.62; N, 4.84. Found: C, 66.10; H, 6.99; N, 4.49.

(1*R,5*S**,6*R**,8*R**)-Ethyl 8-hydroxy-3-phenyl-2-oxa-4-azabicyclo[3.3.1]non-3-ene-6-carboxylate ((±)-9)**

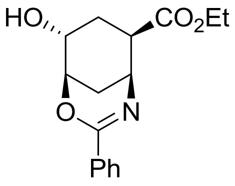

Yellowish oil; yield 64%; R_f = 0.49 (*n*-hexane/EtOAc 1:2). ^{1}H-NMR (400 MHz, $CDCl_3$, TMS): δ = 1.30 (t, *J*=7.12 Hz, 3H, CH_3), 1.80-1.91 (m, 3H, H-9 and H-7), 2.24-2.32 (m, 1H, H-9), 3.00-3.08 (m, 1H, H-6), 4.17-4.29 (m, 3H, OCH_2 and H-8), 4.32-4.38 (m, 1H, H-5), 4.52-4.58 (m, 1H, H-1), 7.31-7.38 (m, 2H, CH-Ar), 7.38-7.46 (m, 1H, CH-Ar), 7.85-7.91 (m, 2H, CH-Ar). ^{13}C NMR (100 MHz, $CDCl_3$, TMS): δ = 14.6, 23.4, 26.7, 43.2, 49.2, 61.1, 68.1, 72.9, 127.7, 128.4, 131.1, 133.7, 157.7, 173.7. MS (ESI, pos) m/z=290 [M+1]. Anal. Calcd. for $C_{16}H_{19}NO_4$: C, 66.42; H, 6.62; N, 4.84. Found: C, 66.11; H, 6.28; N, 4.46.

(1*R,5*S**,6*R**,8*S**)-Ethyl 8-fluoro-3-phenyl-2-oxa-4-azabicyclo[3.3.1]non-3-ene-6-carboxylate ((±)-10)**

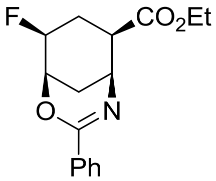

Yellowish oil; yield 18%; R_f = 0.63 (*n*-hexane/EtOAc 3:1). ^{1}H-NMR (400 MHz, $CDCl_3$, TMS): δ = 1.31 (t, *J* = 7.12 Hz, 3H, CH_3), 1.75-2.02 (m, 2H, H-7 and H-9), 2.01-2.23

(m, 2H, H-7 and H-9), 2.93-3.04 (m, 1H, H-6), 4.23 (q, J = 7.12 Hz, 2H, OCH_2), 4.34-4.42 (m, 1H, H-5), 4.70-4.77 (m, 1H, H-1), 4.83-5.03 (m, 1H, H-8), 7.29-7.38 (m, 2H, CH-Ar), 7.38-7.46 (m, 1H, CH-Ar), 7.83-7.92 (m, 2H, CH-Ar). ^{19}F NMR (376 MHz, $CDCl_3$): δ = -185.79. ^{13}C NMR (100 MHz, $CDCl_3$, TMS): δ = 14.6, 23.7, 24.7 and 24.9 (^{2}J = 20.20 Hz), 43.4, 48.8, 61.2, 69.5 and 69.8 (^{2}J = 31.16 Hz), 87.5 and 89.2 (^{1}J = 174.40 Hz), 127.6, 128.4, 131.2, 133.4, 156.2, 172.7. MS (ESI, pos) m/z=292 [M+1]. Anal. Calcd. for $C_{16}H_{18}FNO_3$: C, 65.97; H, 6.23; N, 4.81. Found: C, 65.61; H, 5.88; N, 4.48.

(*S)-Ethyl 6-benzamidocyclohexa-1,3-dienecarboxylate ((±)-11)**

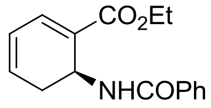

Yellowish oil; yield 26%; R_f = 0.35 (*n*-hexane/EtOAc 2:1). ^{1}H-NMR (400 MHz, $CDCl_3$, TMS): δ = 1.27 (t, J = 7.10 Hz, 3H, CH_3), 2.56-2.68 (m, 1H, H-5), 2.79-2.91 (m, 1H, H-5), 4.15-4.30 (m, 2H, OCH_2), 5.24-5.34 (m, 1H, H-6), 6.10-6.29 (m, 3H, H-3 and H-4 and NH), 7.24-7.31 (m, 1H, H-2), 7.35-7.43 (m, 2H, CH-Ar), 7.43-7.50 (m, 1H, CH-Ar), 7.66-7.74 (m, 2H, CH-Ar). ^{13}C NMR (100 MHz, $CDCl_3$, TMS): δ = 14.6, 31.4, 40.8, 61.2, 123.6, 127.0, 127.3, 128.9, 131.8, 133.6, 135.0, 136.0, 166.6, 167.0. MS (ESI, pos) m/z=272 [M+1]. Anal. Calcd. for $C_{16}H_{17}NO_3$: C, 70.83; H, 6.32; N, 5.16. Found: C, 70.49; H, 5.99; N, 4.80.

(3a*S,4*S**,5*S**,7a*S**)-Ethyl 4-fluoro-2-phenyl-3a,4,5,6,7,7a-hexahydrobenzo-[*d*]oxazole-5-carboxylate ((±)-17)**

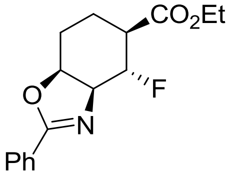

Pale yellow oil; yield 66%; R_f = 0.54 (*n*-hexane/EtOAc 2:1). ^{1}H-NMR (400 MHz, D_6-DMSO, TMS): δ = 1.17 (t, *J* = 7.08 Hz, 3H, CH_3), 1.50-1.64 (m, 1H, H-6), 1.79-2.01 (m, 2H, H-6 and H-7), 2.09-2.20 (m, 1H, H-7), 2.69-2.83 (m, 1H, H-5), 4.09 (q, *J* = 7.08, 2H, OCH_2), 4.18-4.31 (m, 1H, H-3a), 4.46-4.66 (m, 1H, H-4), 4.80-4.89 (m, 1H, H-7a), 7.45-7.53 (m, 2H, CH-Ar), 7.54-7.61 (m, 1H, CH-Ar), 7.84-7.92 (m, 2H, CH-Ar). ^{19}F NMR (376 MHz, D_6-DMSO): δ = -176.72. ^{13}C NMR (100 MHz, D_6-DMSO, TMS): δ = 14.3, 21.0 and 21.1 (^3J = 6.46 Hz), 24.4, 44.5 and 44.7 (^2J = 19.39 Hz), 60.9, 67.4 and 67.7 (^2J = 22.71 Hz), 79.8 and 79.9 (^3J = 8.27 Hz), 94.9 and 96.6 (^1J = 178.56 Hz), 127.6, 128.2, 129.0, 132.2, 165.1, 172.4. MS (ESI, pos) m/z=292 [M+1]. Anal. Calcd. for $C_{16}H_{18}FNO_3$: C, 65.97; H, 6.23; N, 4.81. Found: C, 65.64; H, 5.86; N, 4.49.

(3a*R,4*R**,7*R**,7a*R**)-Ethyl 7-hydroxy-2-phenyl-3a,4,5,6,7,7a-hexahydrobenzo-[*d*]oxazole-4-carboxylate ((±)-16)**

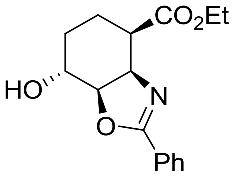

Colorless oil; yield 23%; R_f = 0.39 (*n*-hexane/EtOAc 1:1). ^{1}H-NMR (400 MHz, D_6-DMSO, TMS) δ = 1.18-1.36 (m, 4H, H-6 and CH_3), 1.37-1.52 (m, 1H, H-5), 1.58-1.77

(m, 2H, H-5 and H-6), 2.91-3.02 (m, 1H, H-4), 3.35-3-48 (m, 1H, H-7), 4.15 (q, *J* = 7.02 Hz, 2H, OCH_2), 4.50-4.64 (m, 2H, H-3a and H-7a), 5.20 (d, *J* = 4.48 Hz, 1H, OH), 7.43-7.51 (m, 2H, CH-Ar), 7.51-7.59 (m, 1H, CH-Ar), 7.79-7.88 (m, 2H, CH-Ar). ^{13}C NMR (100 MHz, $CDCl_3$, TMS) δ = 14.7, 20.0, 28.0, 42.6, 61.1, 66.7, 71.7, 84.3, 128.2, 128.6, 128.8, 131.9, 164.8, 173.1. MS (ESI, pos) m/z=290 [M+1]. Anal. Calcd. for $C_{16}H_{19}NO_4$: C, 66.42; H, 6.62; N, 4.84. Found: C, 66.10; H, 6.91; N, 4.55.

(1*S,5*R**,6*R**,9*S**)-Ethyl 9-hydroxy-3-phenyl-2-oxa-4-azabicyclo[3.3.1]non-3-ene-6-carboxylate ((±)-15)**

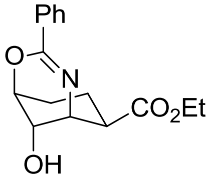

Colorless oil; yield 53%; R_f = 0.40 (*n*-hexane/acetone 2:1). ^{1}H-NMR (400 MHz, D_6-DMSO, TMS) δ = 1.21 (t, *J* = 7.08 Hz, 3H, CH_3), 1.35-1.49 (m, 1H, H-7), 1.55-1.65 (m, 1H, H-7), 1.80-2.09 (m, 2H, H-8), 3.05-3.15 (m, 1H, H-6), 3.91-3.99 (m, 1H, H-9), 3.99-4.15 (m, 3H, OCH_2 and H-5), 4.39-4.46 (m, 1H, H-1), 5.70 (d, *J*=3.24 Hz, 1H, OH), 7.35-7.43 (m, 2H, CH-Ar), 7.43-7.51 (m, 1H, CH-Ar), 7.77-7.85 (m, 2H, CH-Ar). ^{13}C NMR (100 MHz, D_6-DMSO, TMS) δ = 14.5, 17.3, 26.0, 40.7, 55.0, 60.1, 62.6, 73.0, 127.4, 128.5, 131.0, 132.9, 156.1, 173.8. MS (ESI, pos) m/z=290 [M+1]. Anal. Calcd. for $C_{16}H_{19}NO_4$: C, 66.42; H, 6.62; N, 4.84. Found: C, 66.12; H, 6.29; N, 4.52.

(3a*S,4*S**,5*R**,7a*S**)-Ethyl 4-fluoro-2-phenyl-3a,4,5,6,7,7a-hexahydrobenzo-[*d*]oxazole-5-carboxylate ((±)-23)**

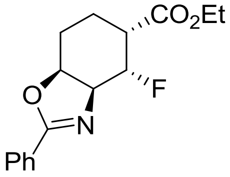

Colorless oil; yield 23%; R_f = 0.41 (*n*-hexane/acetone 6:1). ^{1}H-NMR (400 MHz, $CDCl_3$, TMS): δ = 1.27 (t, *J* = 7.10 Hz, 3H, CH_3),–1.76-1.88 (m, 2H, H-6 and H-7), 1.98-2.08 (m, 1H, H-6), 2.08-2.19 (m, 1H, H-7), 2.59-2.76 (m, 1H, H-5), 4.14-4.26 (m, 2H, OCH_2), 4.50-4.59 (m, 1H, H-3a), 4.94-5.01 (m, 1H, H-7a), 5.38-5.57 (m, 1H, H-4), 7.38-7.46 (m, 2H, CH-Ar), 7.47-7.54 (m, 1H, CH-Ar), 7.91-7.98 (m, 2H, CH-Ar). ^{19}F NMR (376 MHz, $CDCl_3$): δ = -185.23. ^{13}C NMR (100 MHz, $CDCl_3$, TMS): δ = 14.6, 16.0, 24.8, 40.8 and 41.0 (^2J = 21.09 Hz), 61.4, 67.2 and 67.5 (^2J = 28.06 Hz), 77.5, 89.4 and 91.1 (^1J = 173.44 Hz), 127.9, 128.8, 128.8, 132.2, 166.1, 172.3. MS (ESI, pos) m/z=292 [M+1]. Anal. Calcd. for $C_{16}H_{18}FNO_3$: C, 65.97; H, 6.23; N, 4.81. Found: C, 65.59; H, 5.89; N, 4.49.

(3a*R,4*S**,7*S**,7a*R**)-Ethyl 7-fluoro-2-phenyl-3a,4,5,6,7,7a-hexahydrobenzo[*d*]-oxazole-4-carboxylate ((±)-22)**

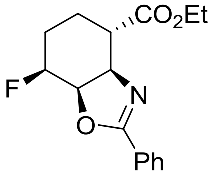

Colorless oil; yield 22%; R_f = 0.35 (*n*-hexane/acetone 2:1). ^{1}H-NMR (400 MHz, $CDCl_3$, TMS) δ = 1.30 (t, *J* = 7.14 Hz, 3H, CH_3), 1.63-1.77 (m, 1H, H-5), 1.80-2.10 (m, 3H, H-5 and H-6), 2.71-2.82 (m, 1H, H-4), 4.22 (q, *J* = 7.14 Hz, 2H, OCH_2), 4.67-

4.83 (m, 2H, H-7a and H-3a), 4.85-5.04 (m, 1H, H-7), 7.36-7.44 (m, 2H, CH-Ar), 7.44-7.52 (m, 1H, CH-Ar), 7.94-8.02 (m, 2H, CH-Ar). ^{19}F NMR (376 MHz, $CDCl_3$) δ = -187.55. ^{13}C NMR (100 MHz, $CDCl_3$, TMS) δ = 14.6, 20.9 and 20.9 (^{3}J=5.27 Hz), 23.7 and 23.9 (^{2}J=20.24 Hz), 43.9, 61.4, 66.7, 77.9 and 78.0 (^{2}J=15.69 Hz), 87.4 and 89.2 (^{1}J=180.17 Hz), 127.8, 128.7, 128.9, 132.0, 165.1, 174.7. MS (ESI, pos) m/z=292 [M+1]. Anal. Calcd. for $C_{16}H_{18}FNO_3$: C, 65.97; H, 6.23; N, 4.81. Found: C, 66.34; H, 5.90; N, 5.19.

(3a*R,4*S**,7*R**,7a*R**)-Ethyl 7-hydroxy-2-phenyl-3a,4,5,6,7,7a-hexahydrobenzo-[*d*]oxazole-4-carboxylate ((±)-21)**

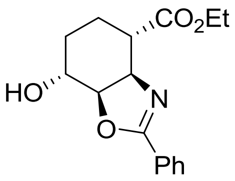

Colorless oil; yield 48%; R_f = 0.32 (*n*-hexane/EtOAc 1:1). ^{1}H-NMR (400 MHz, D_6-DMSO, TMS) δ = 1.21 (t, *J* = 7.10 Hz, 3H, CH_3), 1.35-1.50 (m, 1H, H-6), 1.61-1.84 (m, 3H, H-5 and H-6), 2.53-2.64 (m, 1H, H-4), 3.73-3.84 (m, 1H, H-7), 4.06-4.20 (m, 2H, OCH_2), 4.41-4.52 (m, 2H, H-3a and H-7a), 5.17 (d, *J* = 3.92 Hz, 1H, OH), 7.43-7.52 (m, 2H, CH-Ar), 7.52-7.60 (m, 1H, CH-Ar), 7.83-7.91 (m, 2H, CH-Ar). ^{13}C NMR (100 MHz, D_6-DMSO, TMS) δ = 15.0, 22.0, 27.5, 44.7, 61.1, 66.1, 67.9, 84.3, 128.4, 128.7, 129.4, 132.5, 163.9, 174.7. MS (ESI, pos) m/z=290 [M+1]. Anal. Calcd. for $C_{16}H_{19}NO_4$: C, 66.42; H, 6.62; N, 4.84. Found: C, 66.10; H, 6.43; N, 5.18.

(1*R,2*R**,5*R**,6*R**)-7-Benzyl 2-ethyl 5-hydroxy-7-azabicyclo[4.1.0]heptane-2,7-dicarboxylate ((±)-19)**

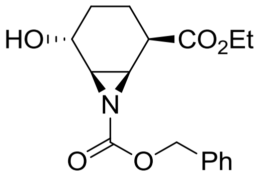

White solid; yield 60%; R_f = 0.28 (*n*-hexane/EtOAc 1:1); Mp 45-50 °C. ^{1}H-NMR (400 MHz, D_6-DMSO, TMS) δ = 1.02-1.17 (m, 4H, CH_3 and H-4), 1.26-1.38 (m, 1H, H-3), 1.46-1.57 (m, 1H, H-3), 1.66-1.77 (m, 1H, H-4), 2.64-2.70 (m, 1H, H-6) vagy 2.77 (d, *J*=6.04 Hz, 1H, H-6), 2.83-2.92 (m, 1H, H-2), 3.05-3.13 (m, 1H, H-1), 3.71-3.81 (m, 1H, H-5), 3.86-4.07 (m, 2H, OCH_2), 5.05 (s, 2H, benzylic CH_2), 5.19 (d, *J* = 4.56 Hz, 1H, OH), 7.30-7.43 (m, 5H, CH-Ar). ^{13}C NMR (100 MHz, $CDCl_3$, TMS) δ = 14.8, 17.8, 29.2, 39.4, 39.5, 43.3, 60.8, 64.7, 68.2, 128.8, 129.0, 129.3, 136.9, 163.0, 173.1. MS (ESI, pos) m/z=342 [M+Na]. Anal. Calcd. for $C_{17}H_{21}NO_5$: C, 63.94; H, 6.63; N, 4.39. Found: C, 63.70; H, 6.34; N, 4.08.

(1*R,2*S**)-Dimethyl 4-oxocyclohexane-1,2-dicarboxylate ((±)-25)**

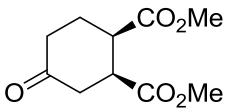

Yellowish oil; yield 48%; R_f = 0.46 (*n*-hexane/EtOAc 1:1). ^{1}H-NMR (400 MHz, $CDCl_3$, TMS): δ = 1.98-2.12 (m, 1H, H-6), 2.27-2.51 (m, 3H, H-6 and H-5), 2.58-2.68 (m, 1H, H-3), 2.82-2.93 (m, 1H, H-3), 3.07-3.16 (m, 1H, H-2), 3.21-3.29 (m, 1 H, H-1), 3.71 (s, 3H, OCH_3), 3.75 (s, 3H, OCH_3). ^{13}C NMR (100 MHz, $CDCl_3$, TMS): δ = 26.1, 38.6, 41.1, 41.6, 43.9, 52.5, 52.6, 172.7, 173.1, 208.3. MS (ESI, pos) m/z=215 [M+1]. Anal. Calcd. for $C_{10}H_{14}O_5$: C, 56.07; H, 6.58; N. Found: C, 56.39; H, 6.13.

X-ray structure determination. The crystal of (±)-**5** was immersed in cryo-oil, mounted in a MiTeGen loop, and measured at 120 K on a Rigaku Oxford Diffraction Supernova diffractometer using CuKα (λ = 1.54184 Å) radiation. The *CrysAlisPro* program package was used for cell refinement and data reduction. An analytical absorption correction (*CrysAlisPro* [1]) was applied to the intensities before structure solution. The structure was solved by intrinsic phasing method using *SHELXT* software. Structural refinement was carried out using *SHELXL-2016* [2]. The O-H and N-H hydrogen atoms were located from the difference Fourier map and refined isotropically. All other H-atoms were positioned geometrically and constrained to ride on their parent atoms, with C-H = 0.95-1.00 Å and U_{iso} = 1.2-1.5·U_{eq} (parent atom). The crystallographic details are summarized in Table S1.

Table S1: Crystal data.

	**(±)-5**
empirical formula	$C_{15}H_{18}FNO_4$
fw	295.30
temp (K)	120(2)
λ(Å)	1.54184
cryst syst	Triclinic
space group	P $\bar{1}$
a (Å)	9.9374(3)
b (Å)	11.1472(3)
c (Å)	14.1887(3)
α(°)	104.542(2)
β(°)	102.666(2)

γ(°)	103.362(2)
V (Å^3)	1414.59(7)
Z	4
ρ_{calc} (Mg/m^3)	1.387
μ(Kα) (mm^{-1})	0.918
No. reflns.	34633
Unique reflns.	5943
GOOF (F^2)	1.034
R_{int}	0.0290
R1^a ($I \geq 2\sigma$)	0.0343
wR2^b ($I \geq 2\sigma$)	0.0905

^{a} $R1 = \Sigma||F_o| - |F_c||/\Sigma|F_o|$. ^{b} $wR2 = [\Sigma[w(F_o^2 - F_c^2)^2]/ \Sigma[w(F_o^2)^2]]^{1/2}$.

References

1. *CrysAlisPro, Revision 5.2;* Agilent Technologies, Inc: Oxfordshire, U.K., 2013.
2. Sheldrick, G. M. *Acta Crystallogr., Sect. C: Struct. Chem.* **2015,** *71,* 3. doi:10.1107/S2053229614024218

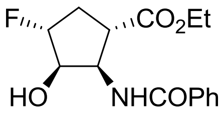
F
CO2Et
HO
NHCOPh

(±)-5

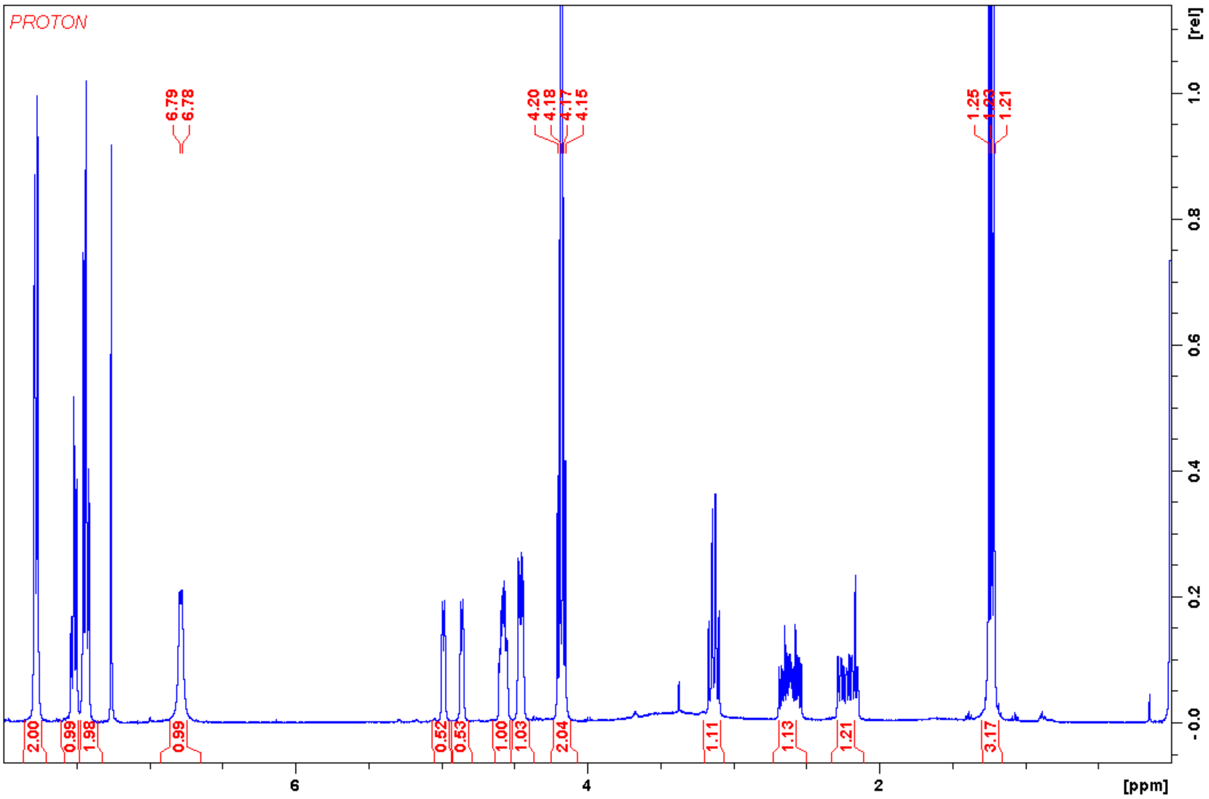
PROTON
6.79
6.78
4.20
4.18
4.17
4.15
1.25
1.21
2.00
0.99
1.98
0.99
0.52
0.53
1.00
1.03
2.04
1.11
1.13
1.21
3.17
6
4
2
[ppm]
[rel]

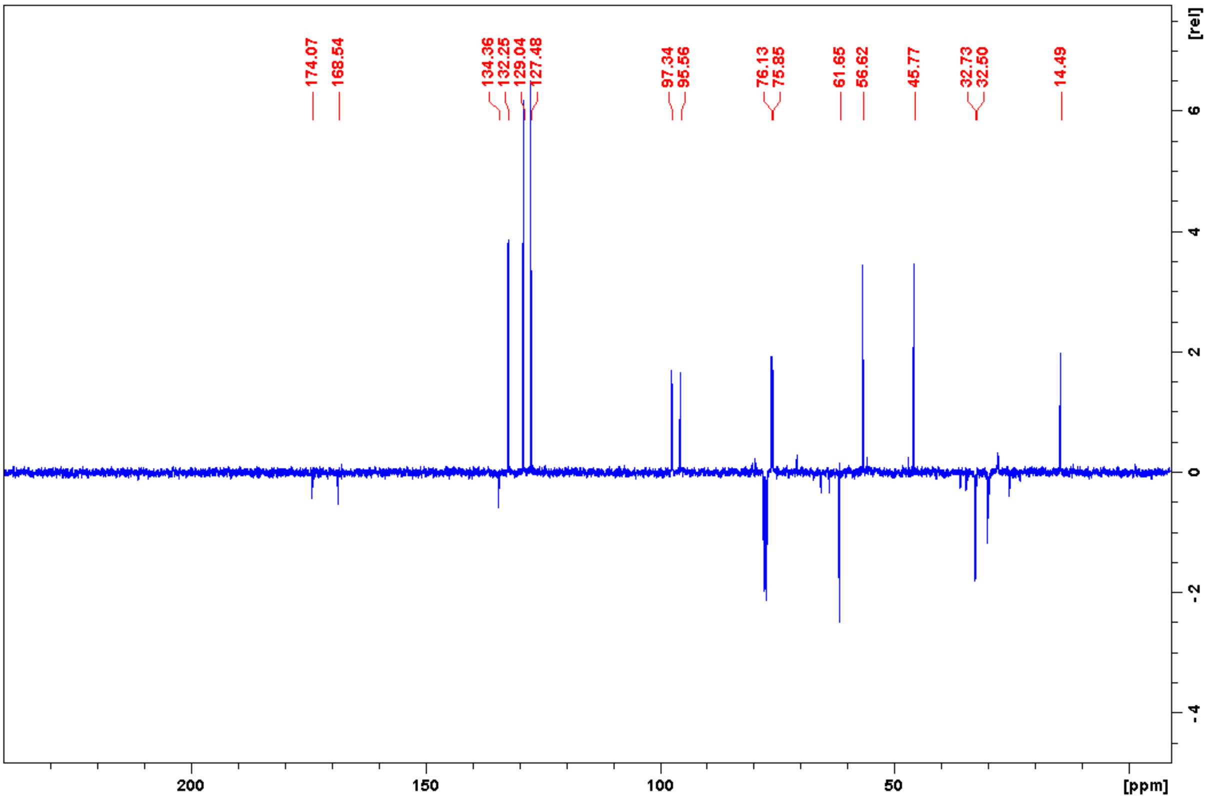
174.07
168.54
134.36
132.25
129.04
127.48
97.34
95.56
76.13
75.85
61.65
56.62
45.77
32.73
32.50
14.49
200
150
100
50
[ppm]
[rel]

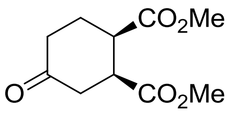

CO2Me
CO2Me
O

(±)-25

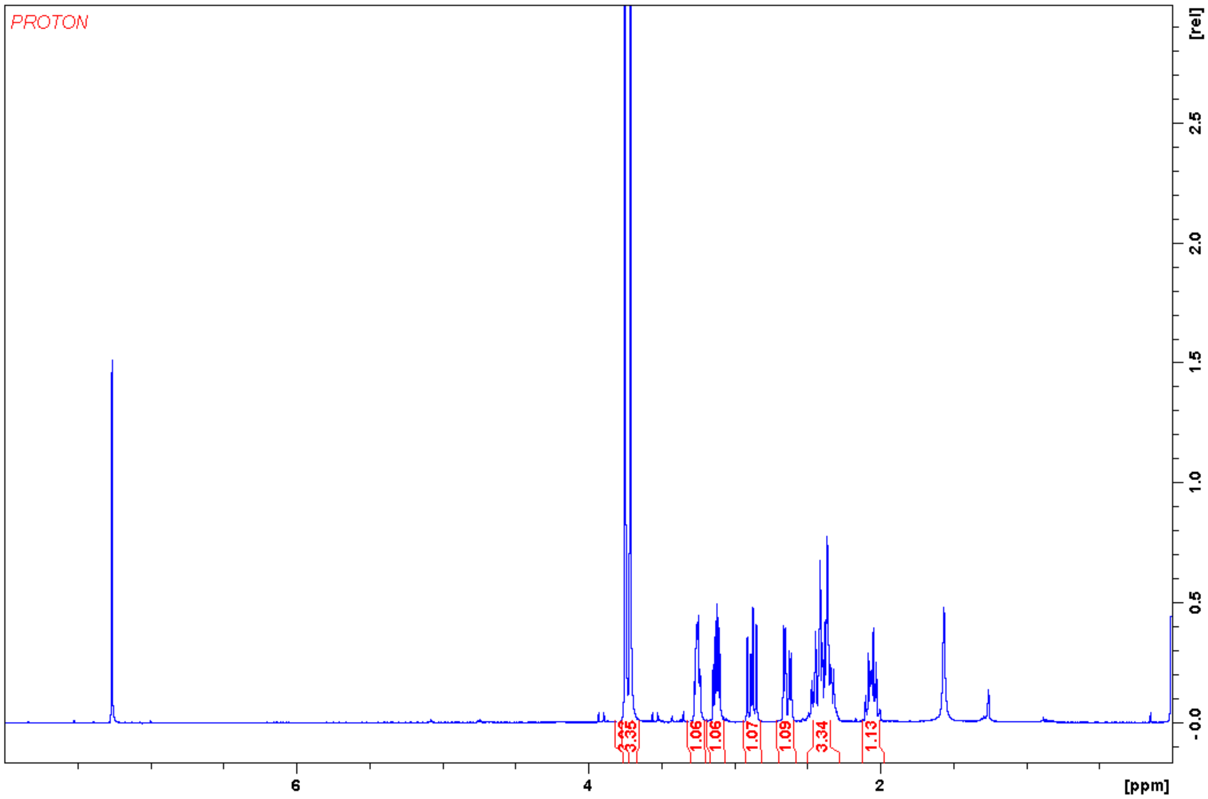

PROTON
3.35
3.35
1.06
1.06
1.07
1.09
3.34
1.13
6
4
2
[ppm]
[rel]
2.5
2.0
1.5
1.0
0.5
-0.0

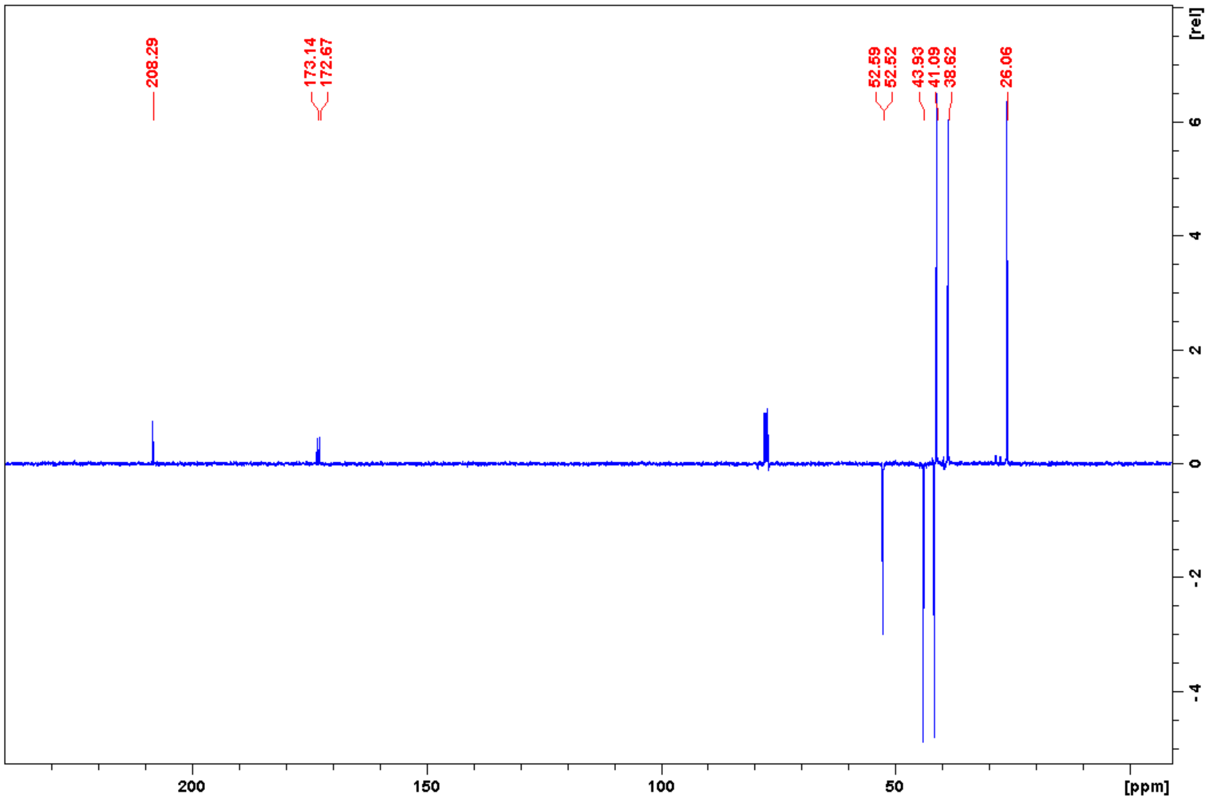

208.29
173.14
172.67
52.59
52.52
43.93
41.09
38.62
26.06
200
150
100
50
[ppm]
[rel]
6
4
2
0
-2
-4

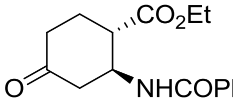
CO2Et
O
NHCOPh

(±)-13

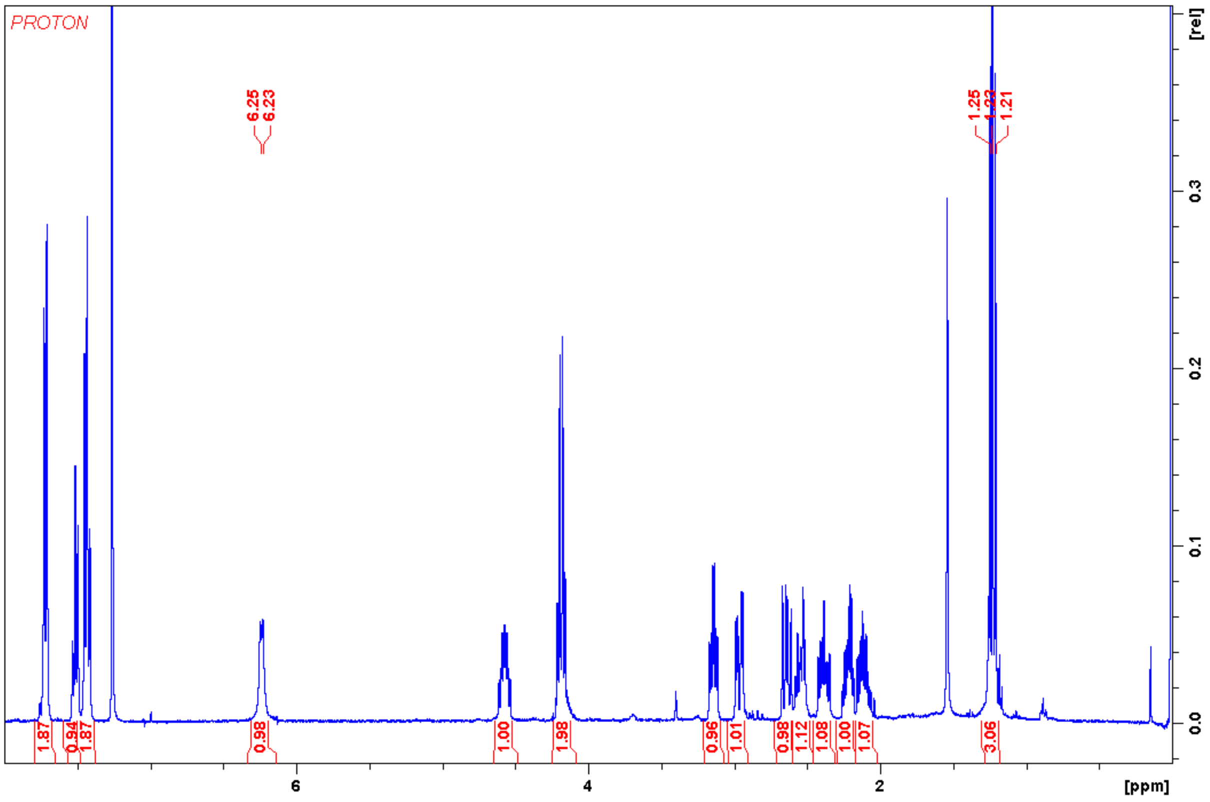
PROTON
6.25
6.23
1.25
1.23
1.21
1.87
0.94
1.87
0.98
1.00
1.98
0.96
1.01
0.98
1.12
1.08
1.00
1.07
3.06
6
4
2
[ppm]
[rel]
0.0
0.1
0.2
0.3

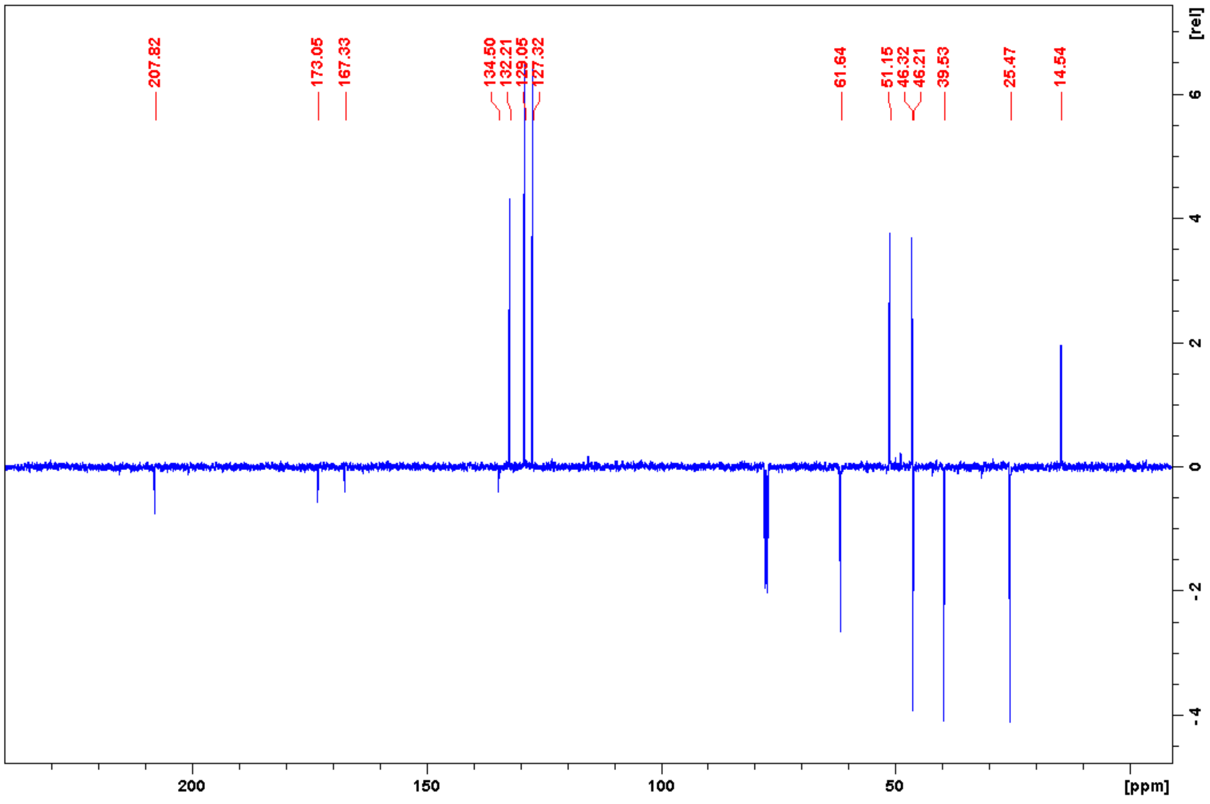
207.82
173.05
167.33
134.50
132.21
129.05
127.32
61.64
51.15
46.32
46.21
39.53
25.47
14.54
200
150
100
50
[ppm]
[rel]
6
4
2
0
-2
-4

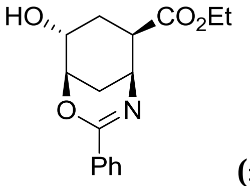
HO
CO2Et
O
N
Ph

(±)-9

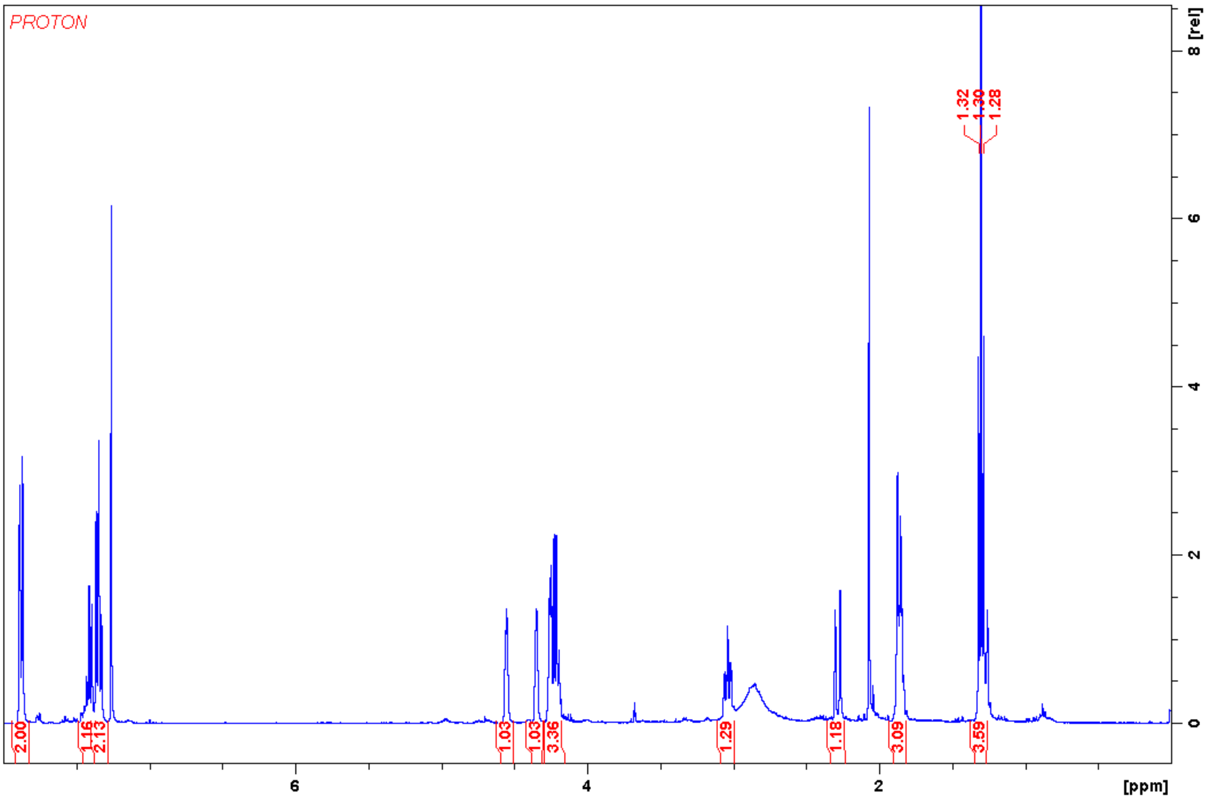
PROTON
1.32
1.30
1.28
2.00
1.16
2.13
1.03
1.03
3.36
1.29
1.18
3.09
3.59
6
4
2
[ppm]
[rel]
8
6
4
2
0

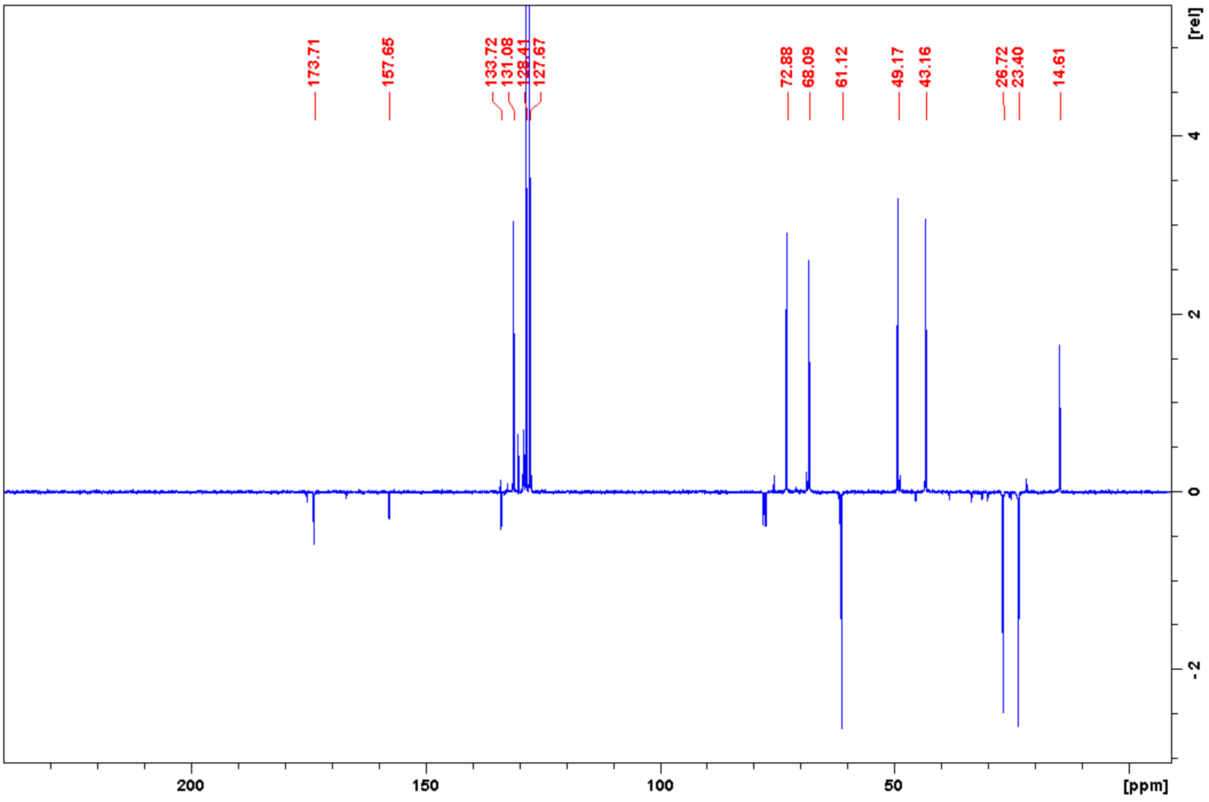
173.71
157.65
133.72
131.08
128.41
127.67
72.88
68.09
61.12
49.17
43.16
26.72
23.40
14.61
200
150
100
50
[ppm]
[rel]
4
2
0
-2

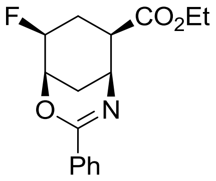

(±)-10

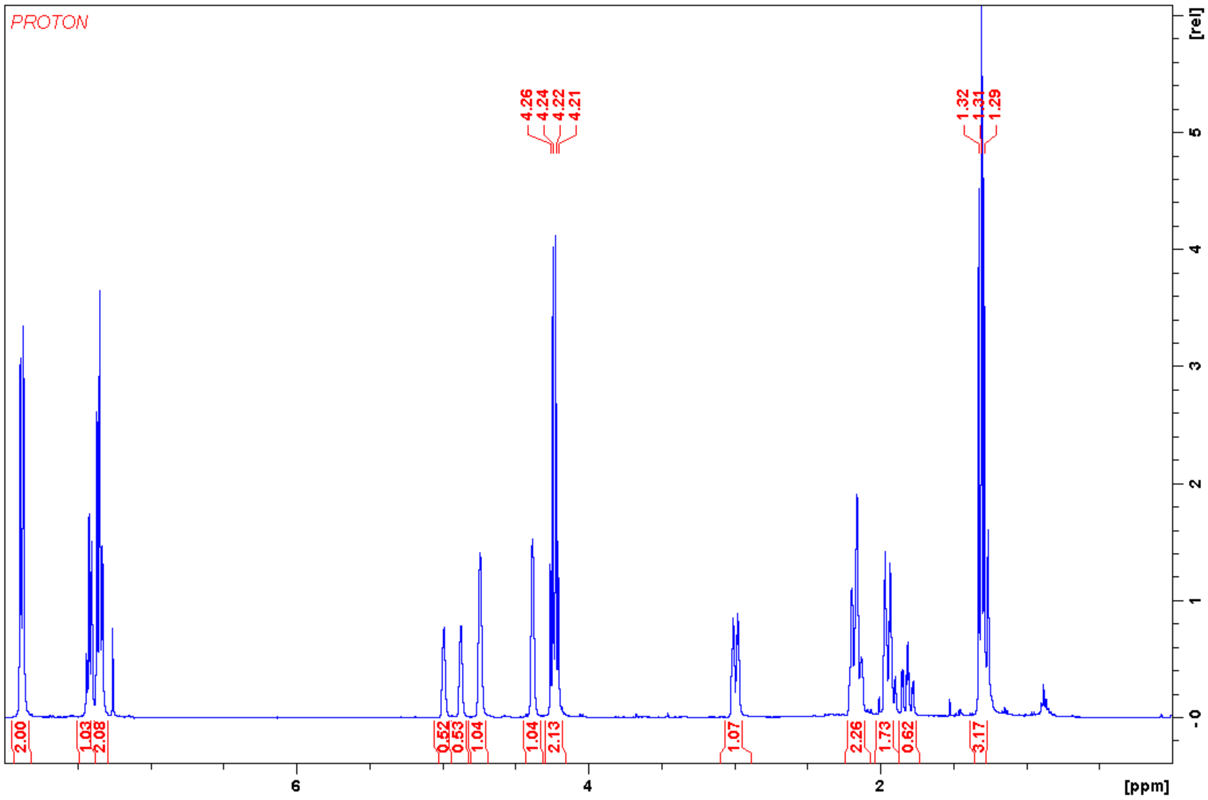
PROTON
4.26
4.24
4.22
4.21
1.32
1.31
1.29
2.00
1.03
2.08
0.52
0.53
1.04
1.04
2.13
1.07
2.26
1.73
0.62
3.17
6
4
2
[ppm]
[rel]

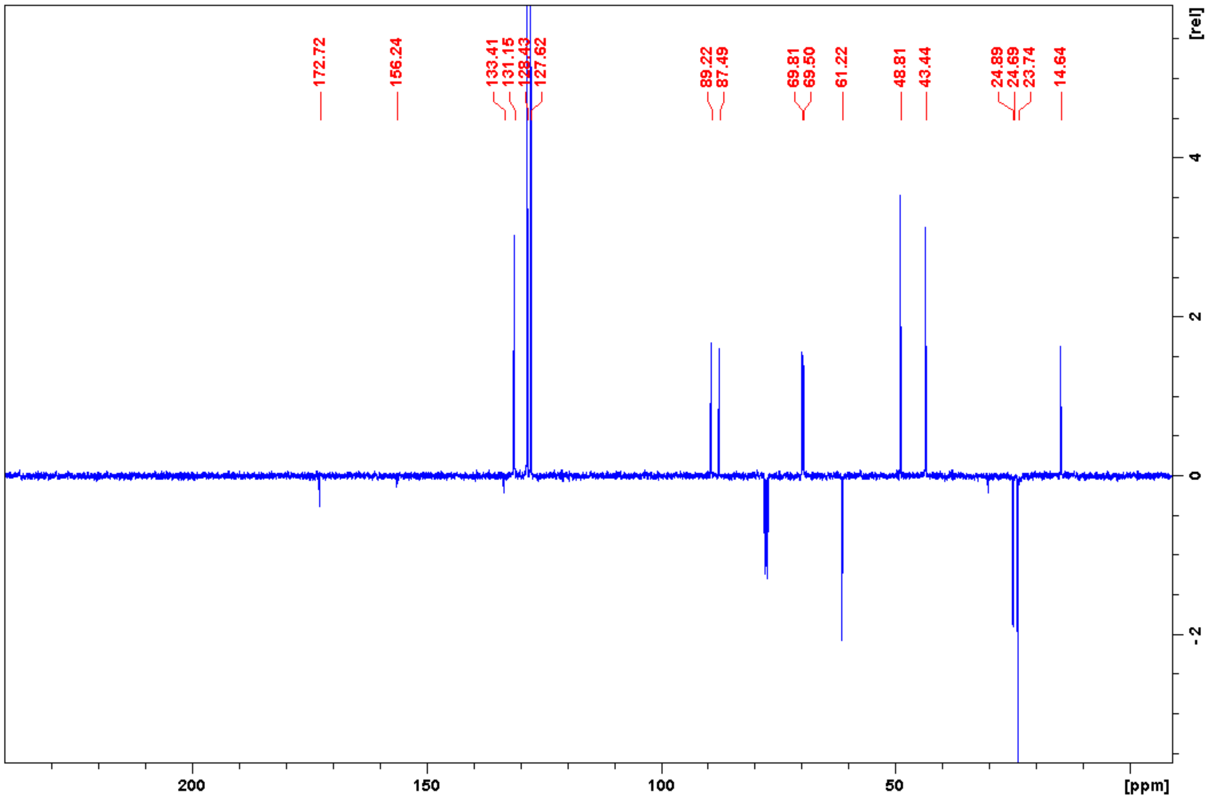
172.72
156.24
133.41
131.15
128.43
127.62
89.22
87.49
69.81
69.50
61.22
48.81
43.44
24.89
24.69
23.74
14.64
200
150
100
50
[ppm]
[rel]

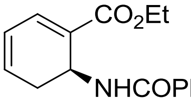
CO2Et
NHCOPh

(±)-11

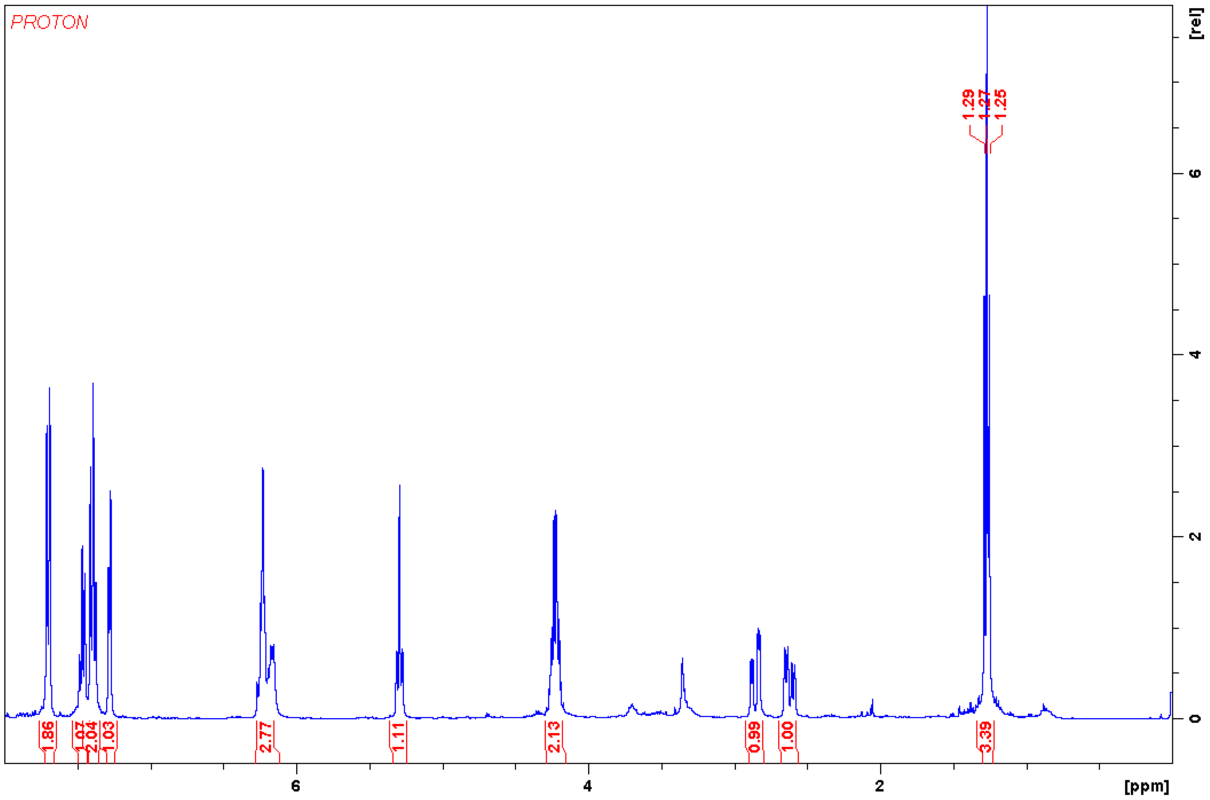
PROTON
1.29
1.27
1.25
1.86
1.07
2.04
1.03
2.77
1.11
2.13
0.99
1.00
3.39
6
4
2
[ppm]
[rel]

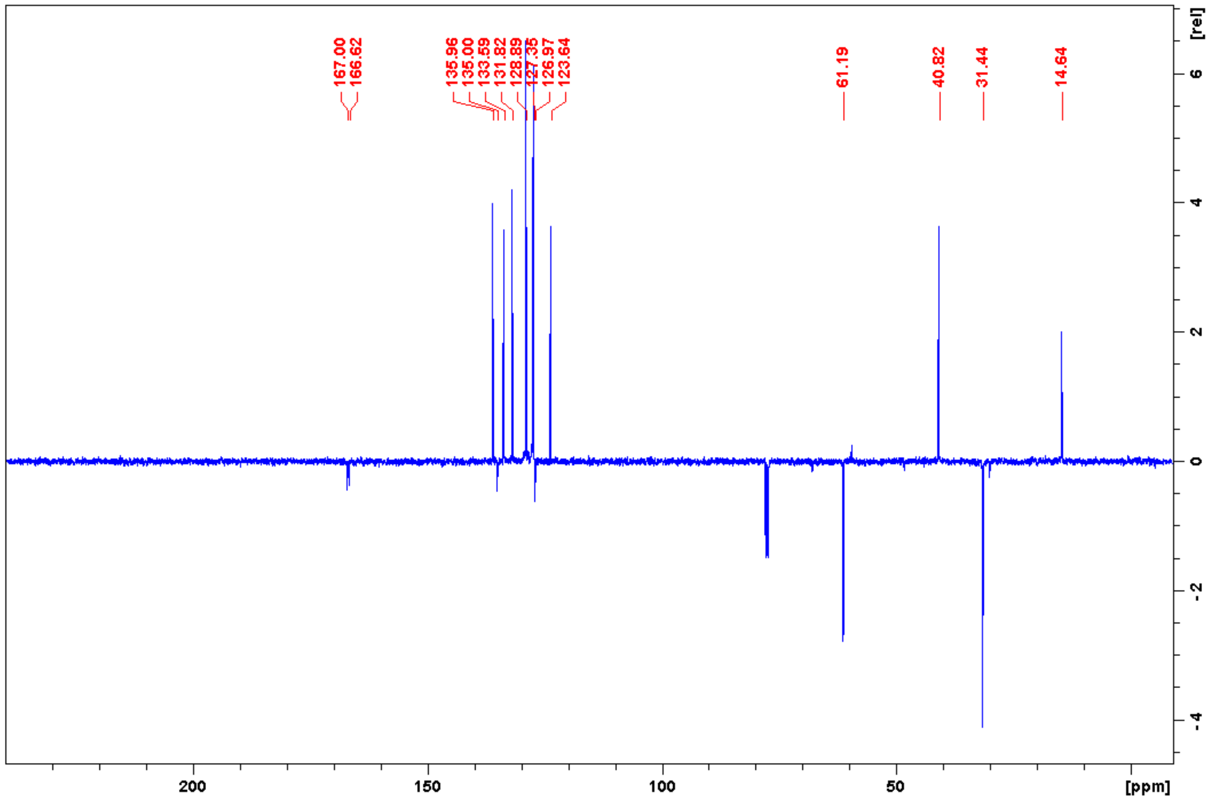
167.00
166.62
135.96
135.00
133.59
131.82
128.89
127.35
126.97
123.64
61.19
40.82
31.44
14.64
200
150
100
50
[ppm]
[rel]

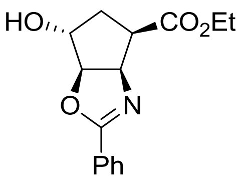
HO
CO2Et
O
N
Ph

(±)-2

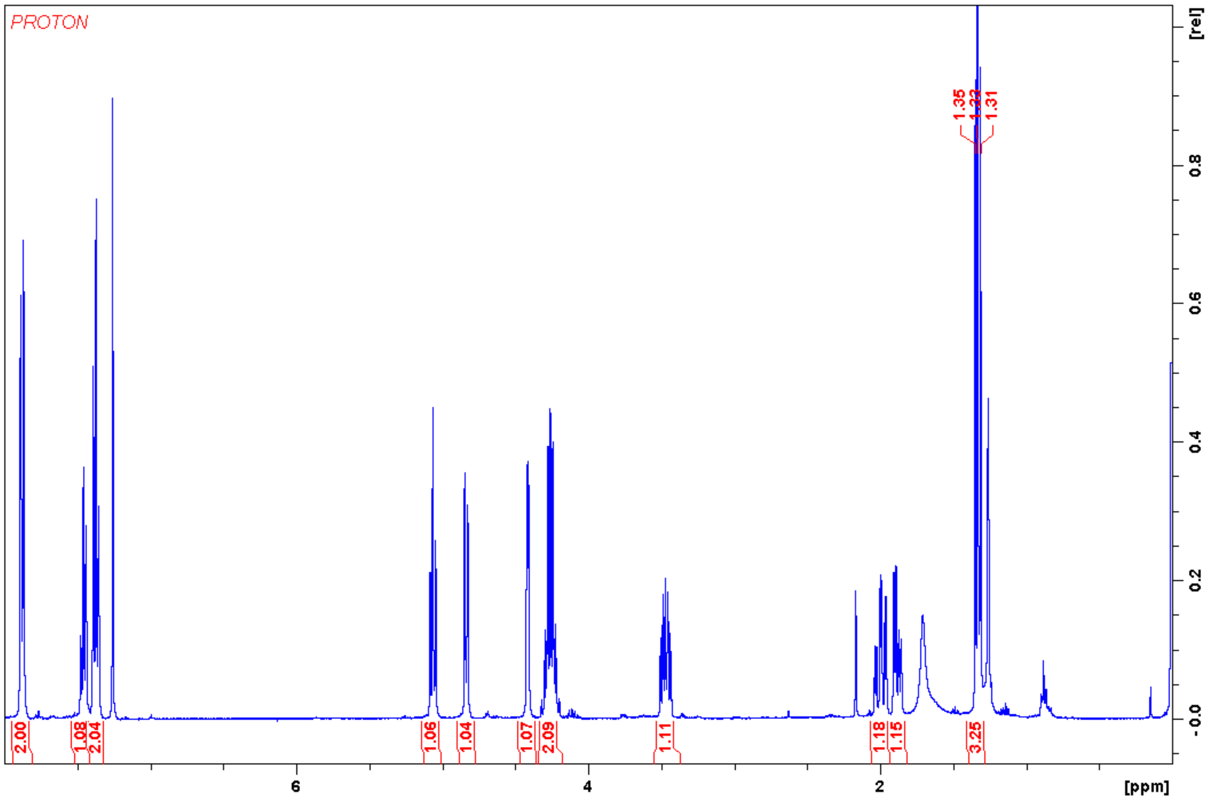
PROTON
1.35
1.33
1.31
2.00
1.08
2.04
1.06
1.04
1.07
2.09
1.11
1.18
1.15
3.25
[rel]
0.8
0.6
0.4
0.2
-0.0
6
4
2
[ppm]

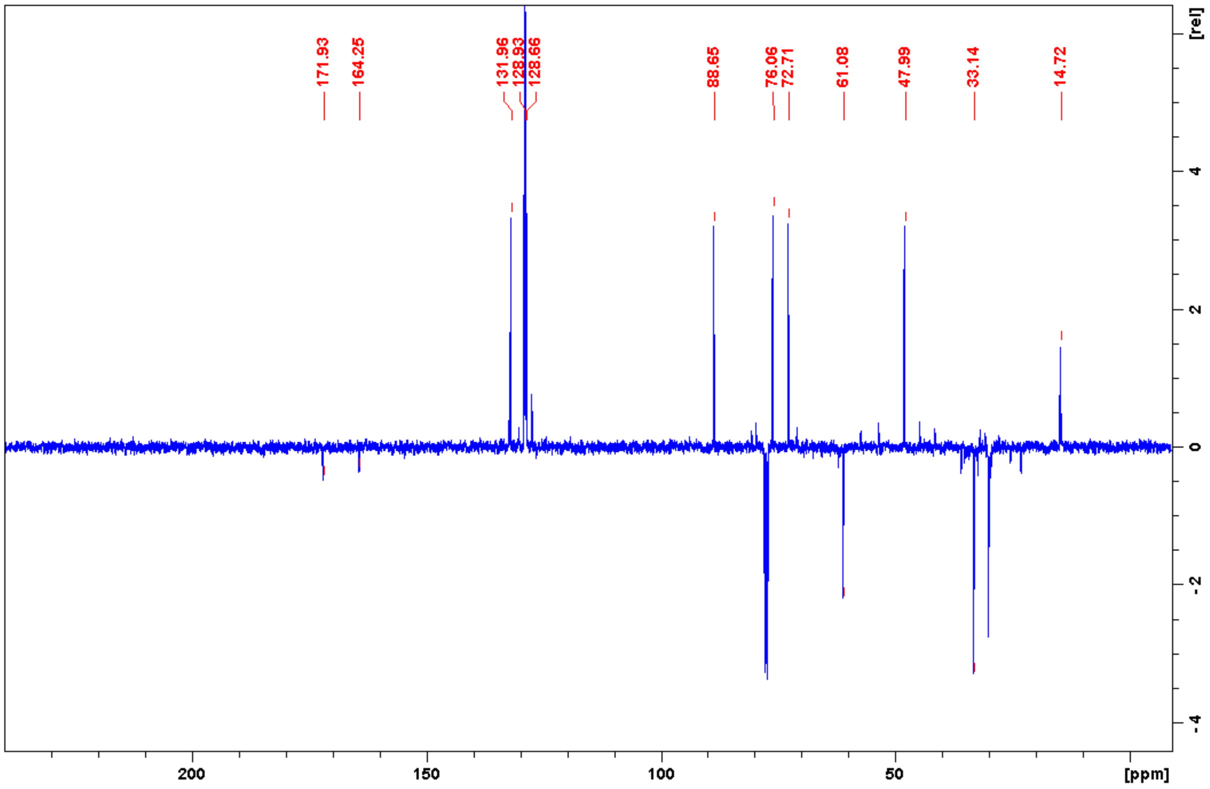
171.93
164.25
131.96
128.93
128.66
88.65
76.06
72.71
61.08
47.99
33.14
14.72
[rel]
4
2
0
-2
-4
200
150
100
50
[ppm]

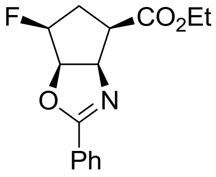

(±)-3

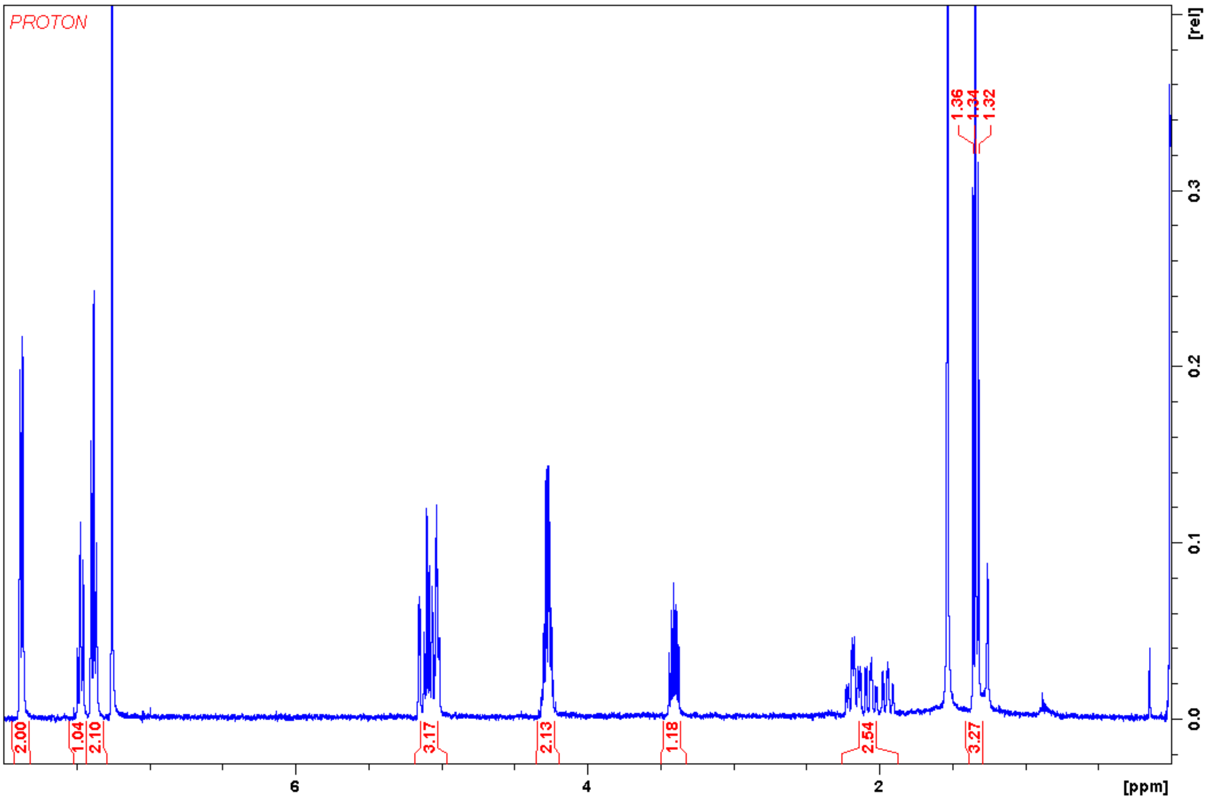
PROTON
1.36
1.34
1.32
2.00
1.04
2.10
3.17
2.13
1.18
2.54
3.27
[ppm]
[rel]

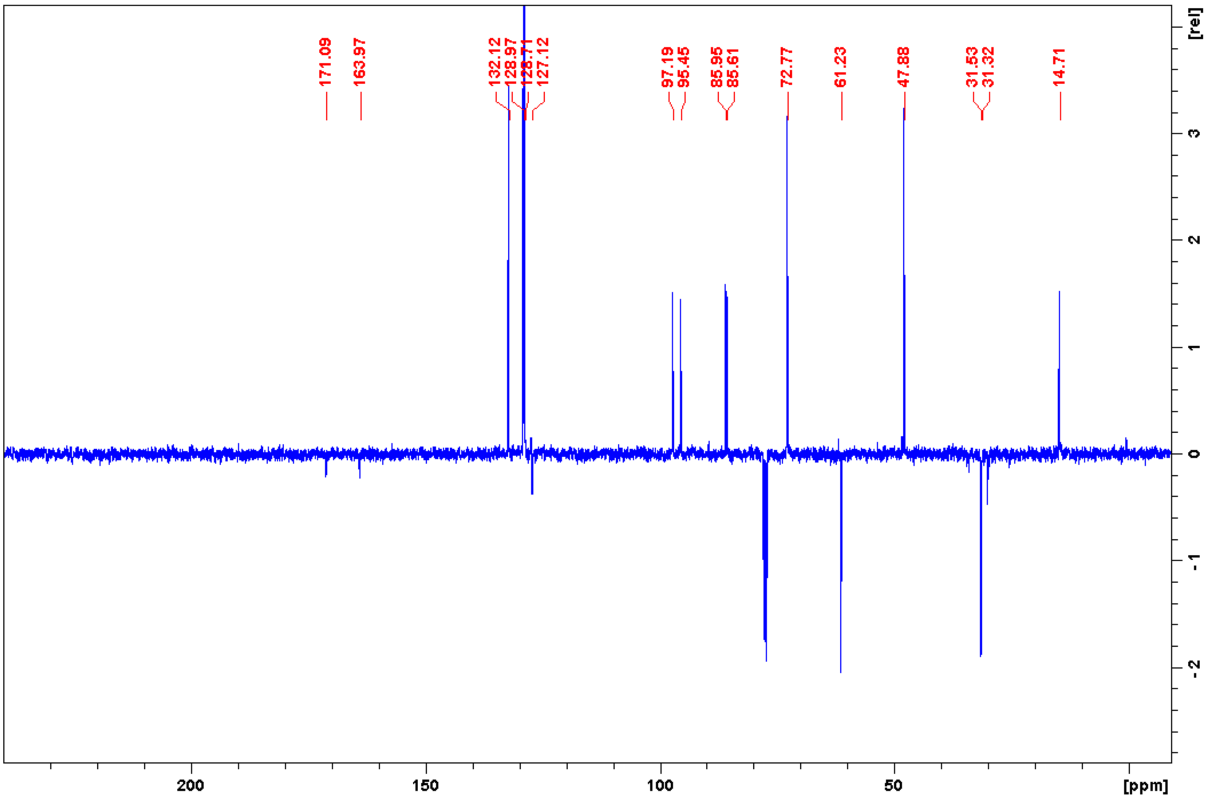
171.09
163.97
132.12
128.97
128.71
127.12
97.19
95.45
85.95
85.61
72.77
61.23
47.88
31.53
31.32
14.71
[ppm]
[rel]

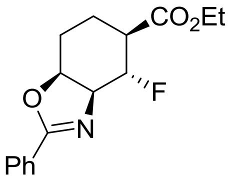
CO2Et
F
O
N
Ph

(±)-17

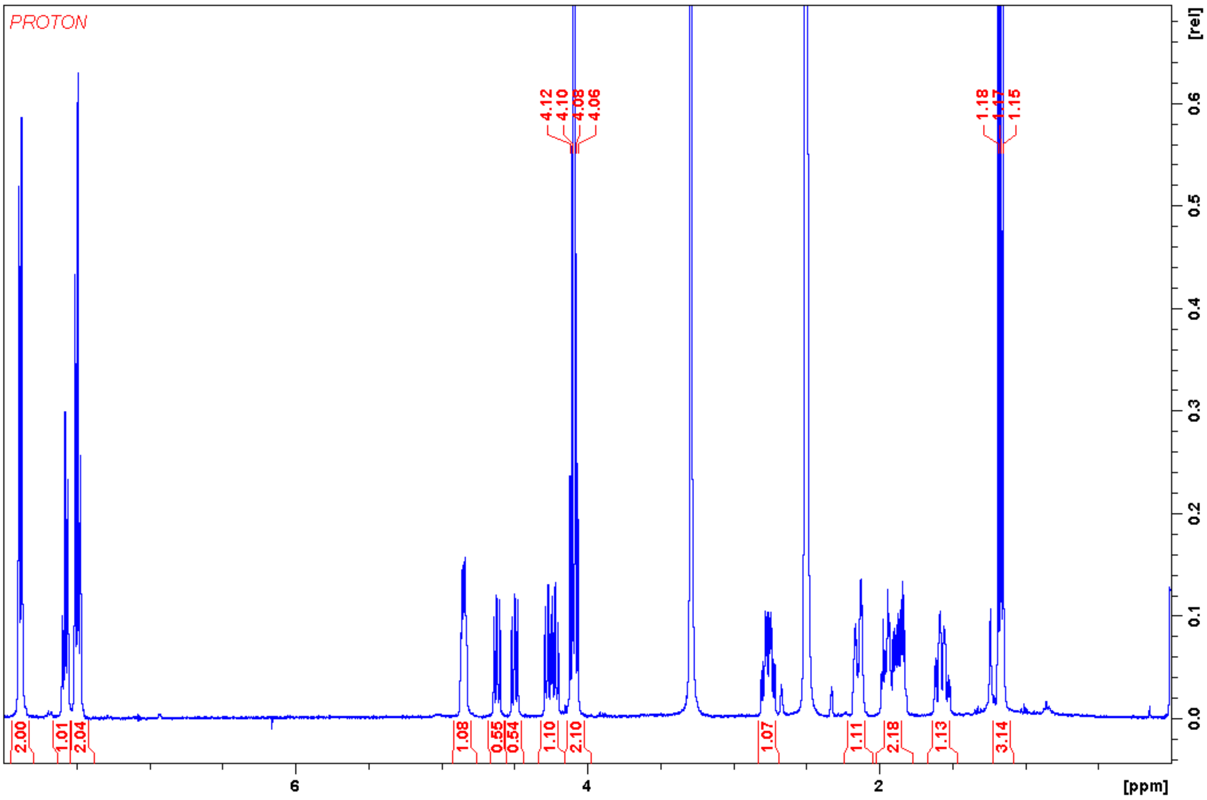
PROTON
4.12
4.10
4.08
4.06
1.18
1.17
1.15
2.00
1.01
2.04
1.08
0.55
0.54
1.10
2.10
1.07
1.11
2.18
1.13
3.14
6
4
2
[ppm]
[rel]

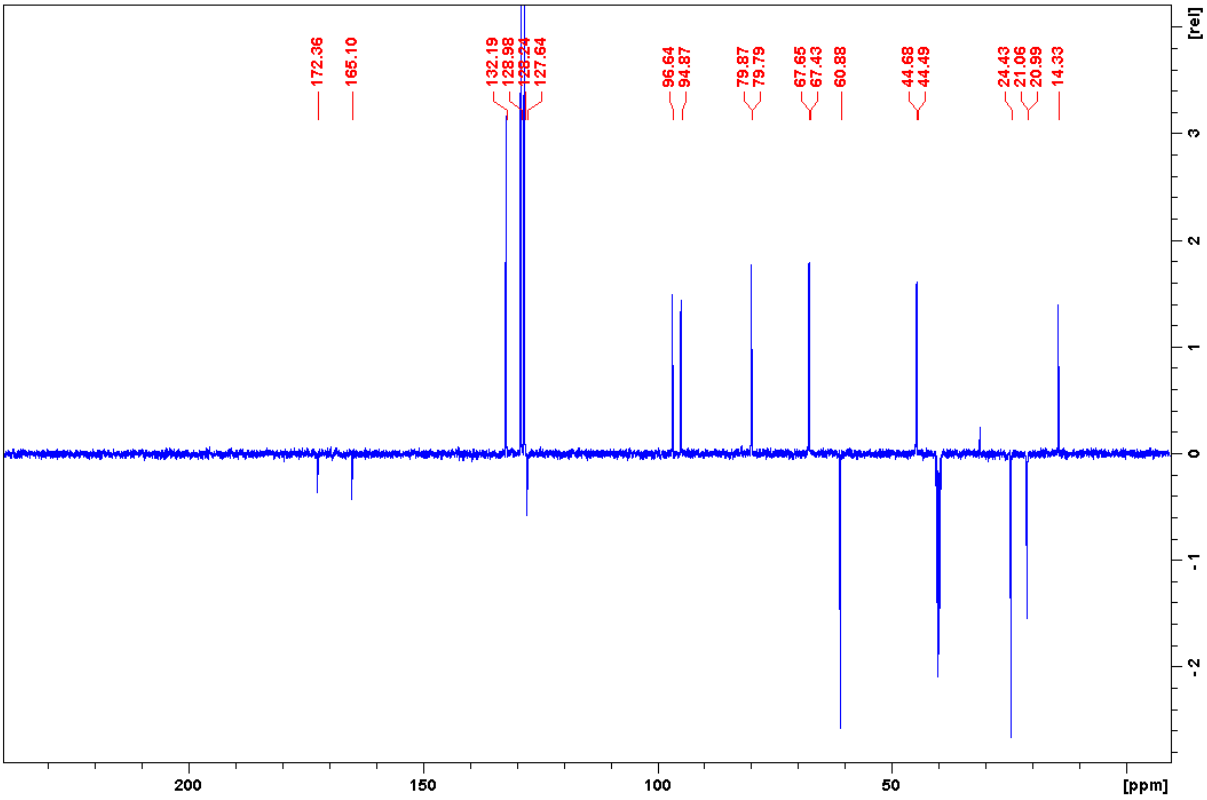
172.36
165.10
132.19
128.98
128.24
127.64
96.64
94.87
79.87
79.79
67.65
67.43
60.88
44.68
44.49
24.43
21.06
20.99
14.33
200
150
100
50
[ppm]
[rel]

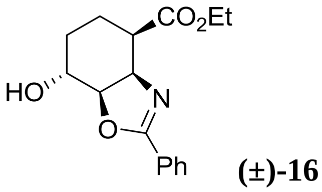
CO2Et
HO
N
O
Ph

(±)-16

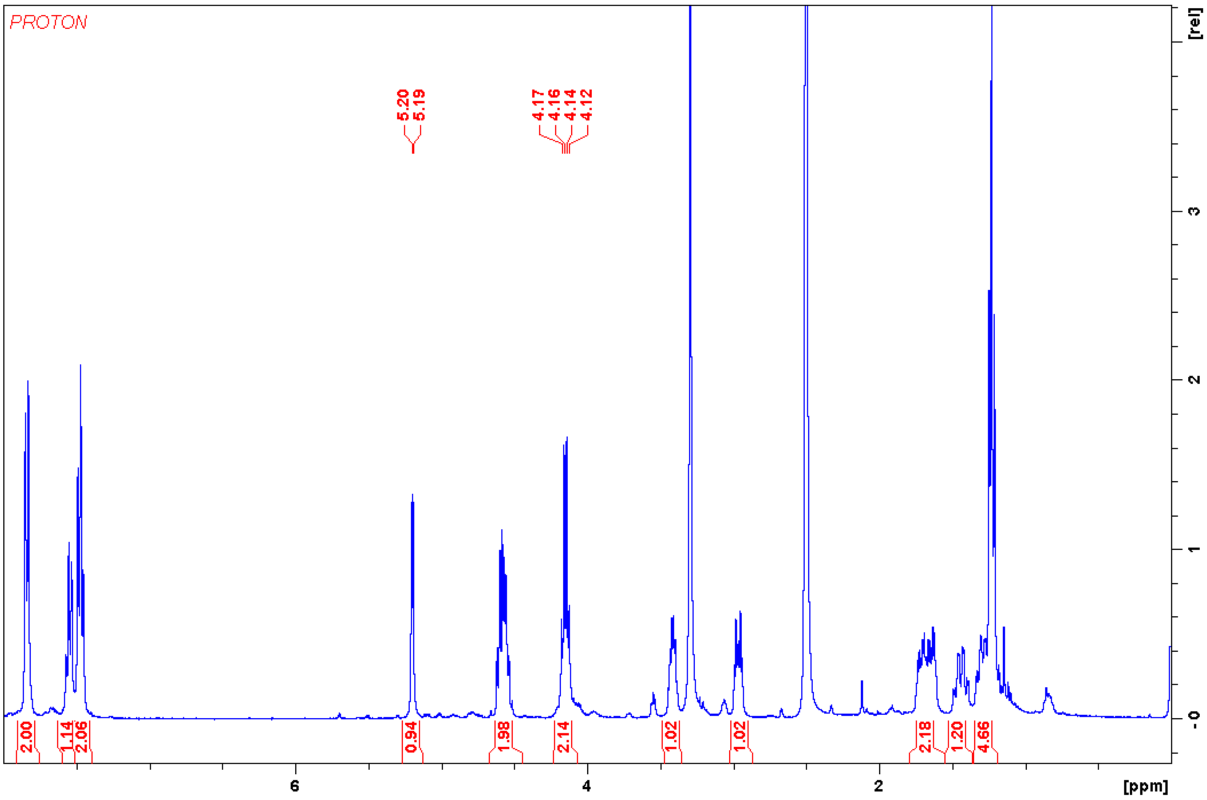
PROTON
5.20
5.19
4.17
4.16
4.14
4.12
2.00
1.14
2.06
0.94
1.98
2.14
1.02
1.02
2.18
1.20
4.66
6
4
2
[ppm]
[rel]

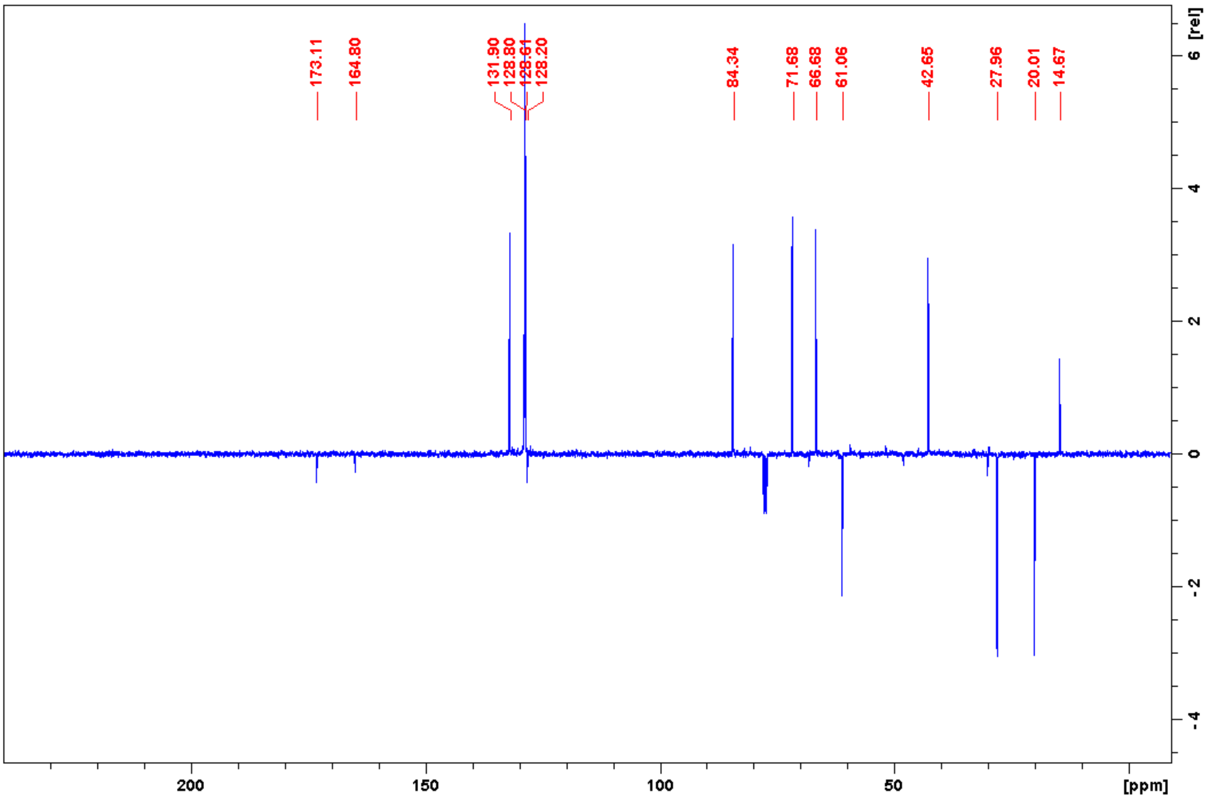
173.11
164.80
131.90
128.80
128.61
128.20
84.34
71.68
66.68
61.06
42.65
27.96
20.01
14.67
200
150
100
50
[ppm]
[rel]

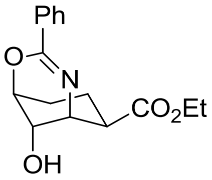
Ph
O
N
CO2Et
OH

(±)-15

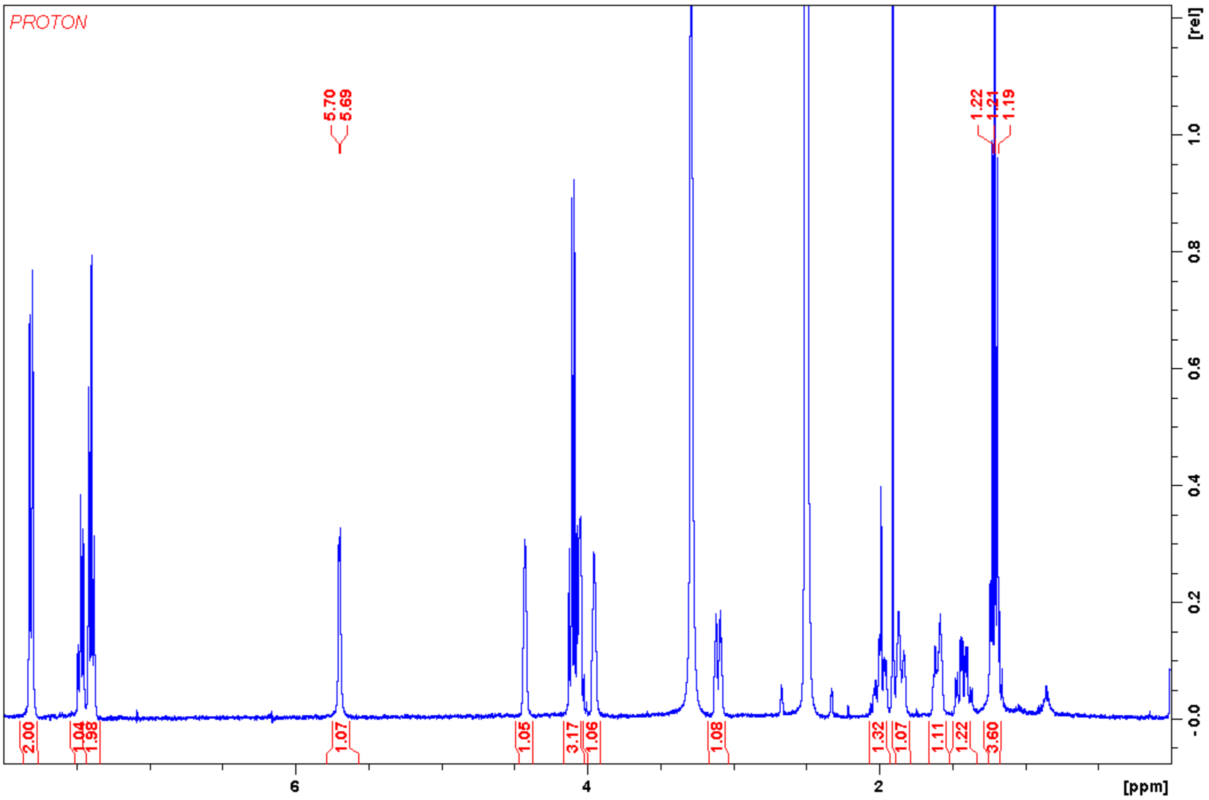
PROTON
5.70
5.69
1.22
1.21
1.19
2.00
1.04
1.98
1.07
1.05
3.17
1.06
1.08
1.32
1.07
1.11
1.22
3.60
[rel]
1.0
0.8
0.6
0.4
0.2
-0.0
6
4
2
[ppm]

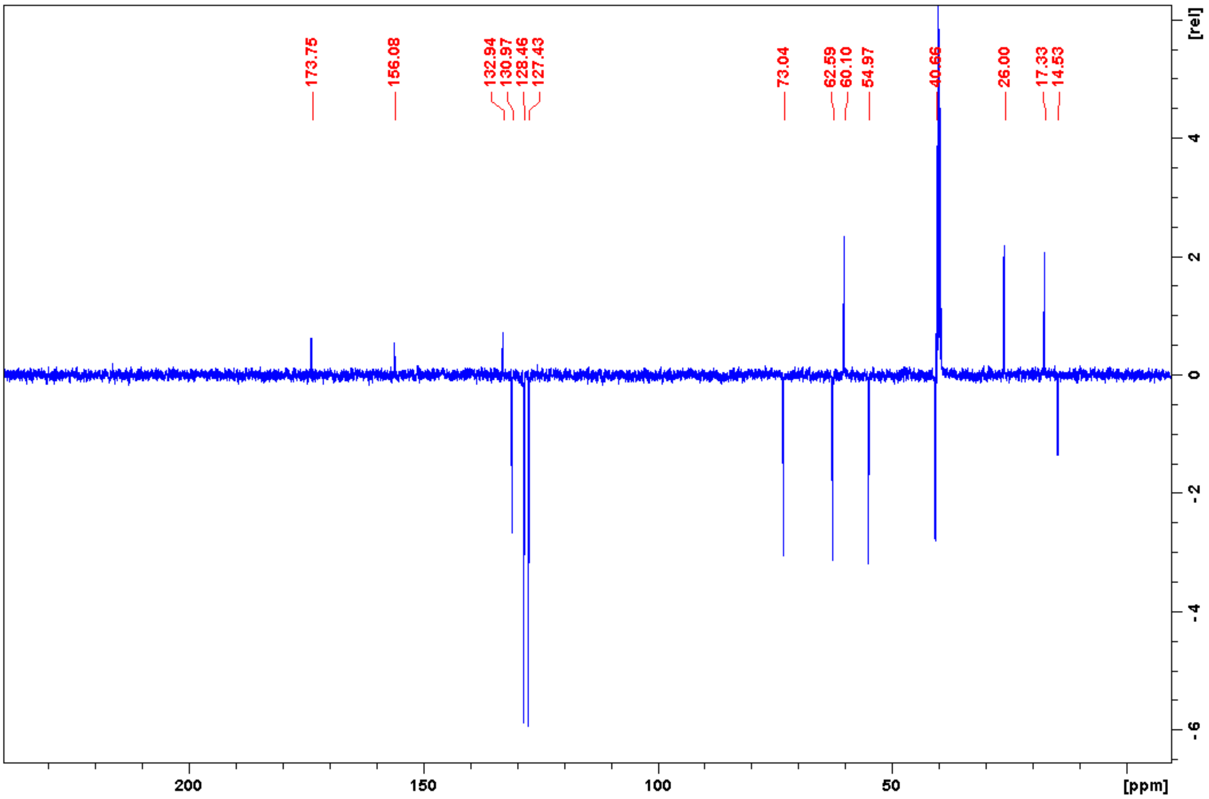
173.75
156.08
132.94
130.97
128.46
127.43
73.04
62.59
60.10
54.97
40.66
26.00
17.33
14.53
[rel]
4
2
0
-2
-4
-6
200
150
100
50
[ppm]

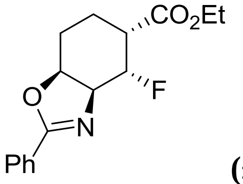
CO2Et
F
O
N
Ph

(±)-23

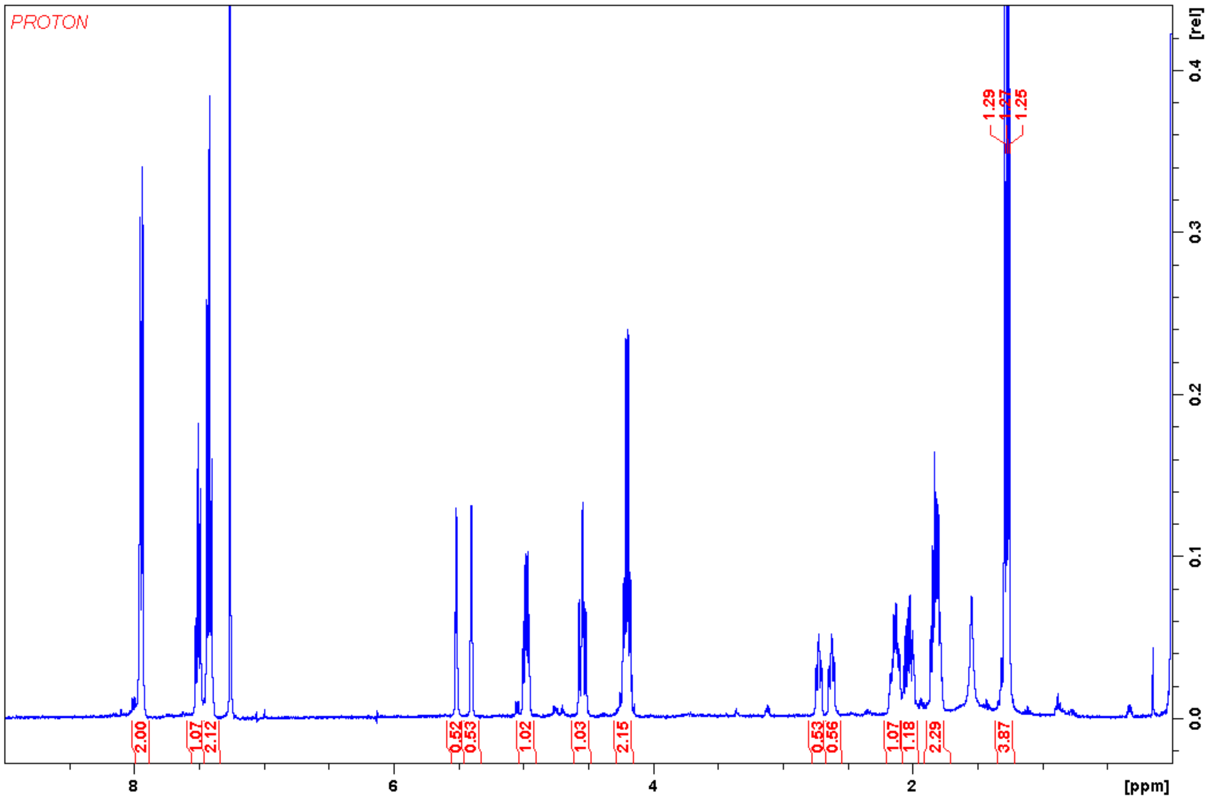
PROTON
1.29
1.27
1.25
2.00
1.07
2.12
0.52
0.53
1.02
1.03
2.15
0.53
0.56
1.07
1.18
2.29
3.87
[ppm]
[rel]

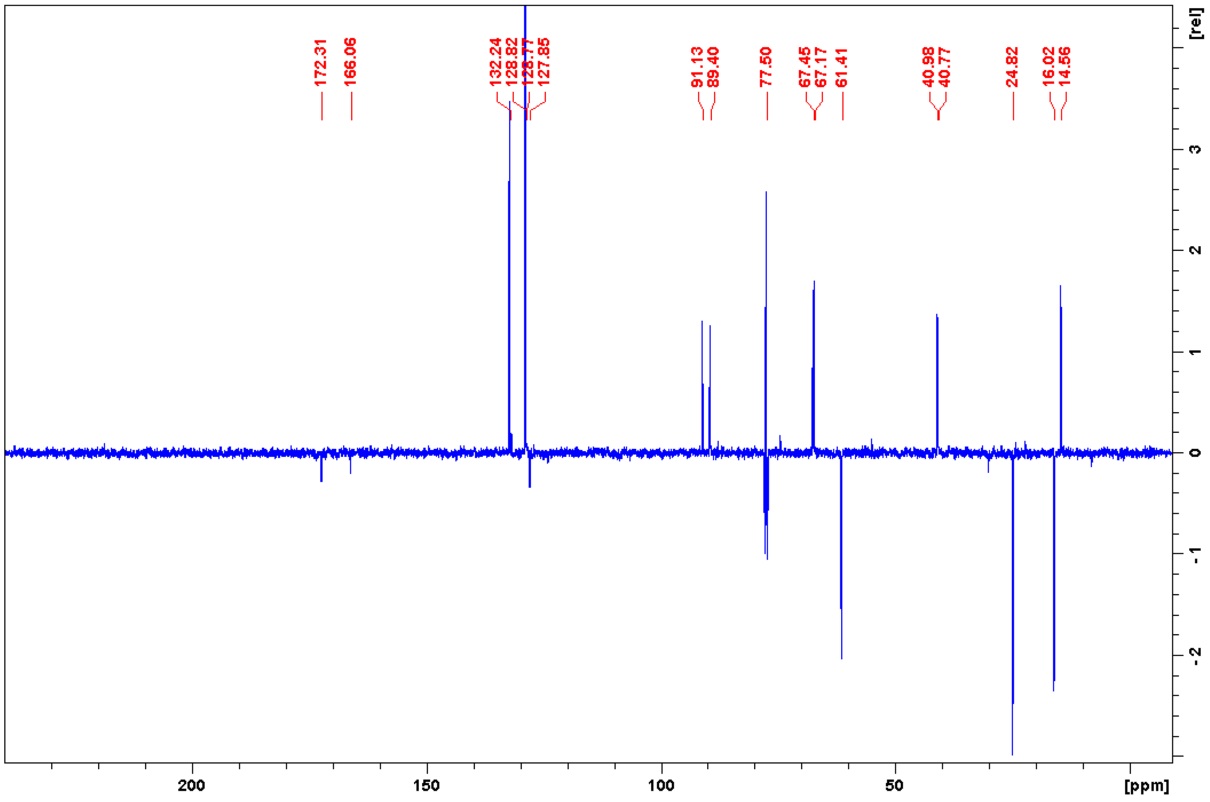
172.31
166.06
132.24
128.82
128.77
127.85
91.13
89.40
77.50
67.45
67.17
61.41
40.98
40.77
24.82
16.02
14.56
[ppm]
[rel]

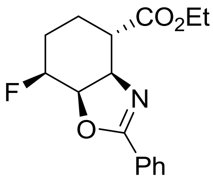
CO2Et
F
N
O
Ph

(±)-22

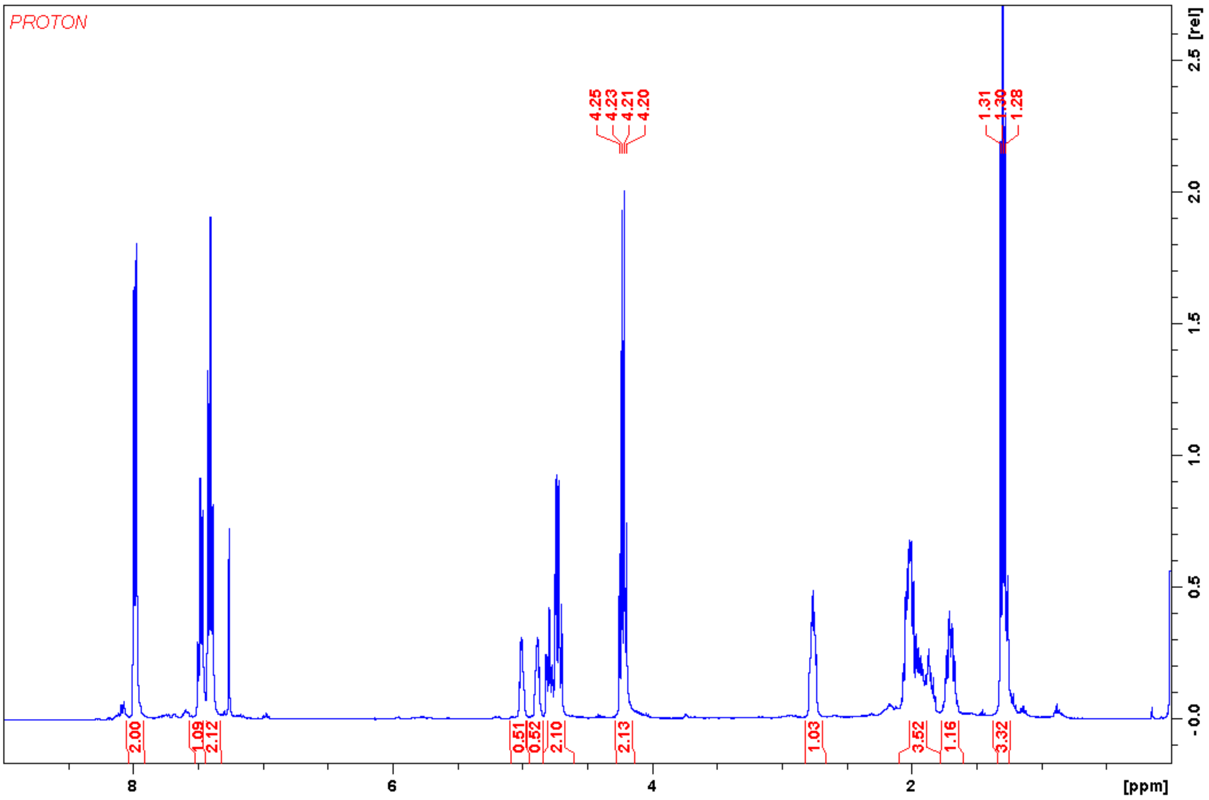
PROTON
4.25
4.23
4.21
4.20
1.31
1.30
1.28
2.00
1.09
2.12
0.51
0.52
2.10
2.13
1.03
3.52
1.16
3.32
8
6
4
2
[ppm]
[rel]

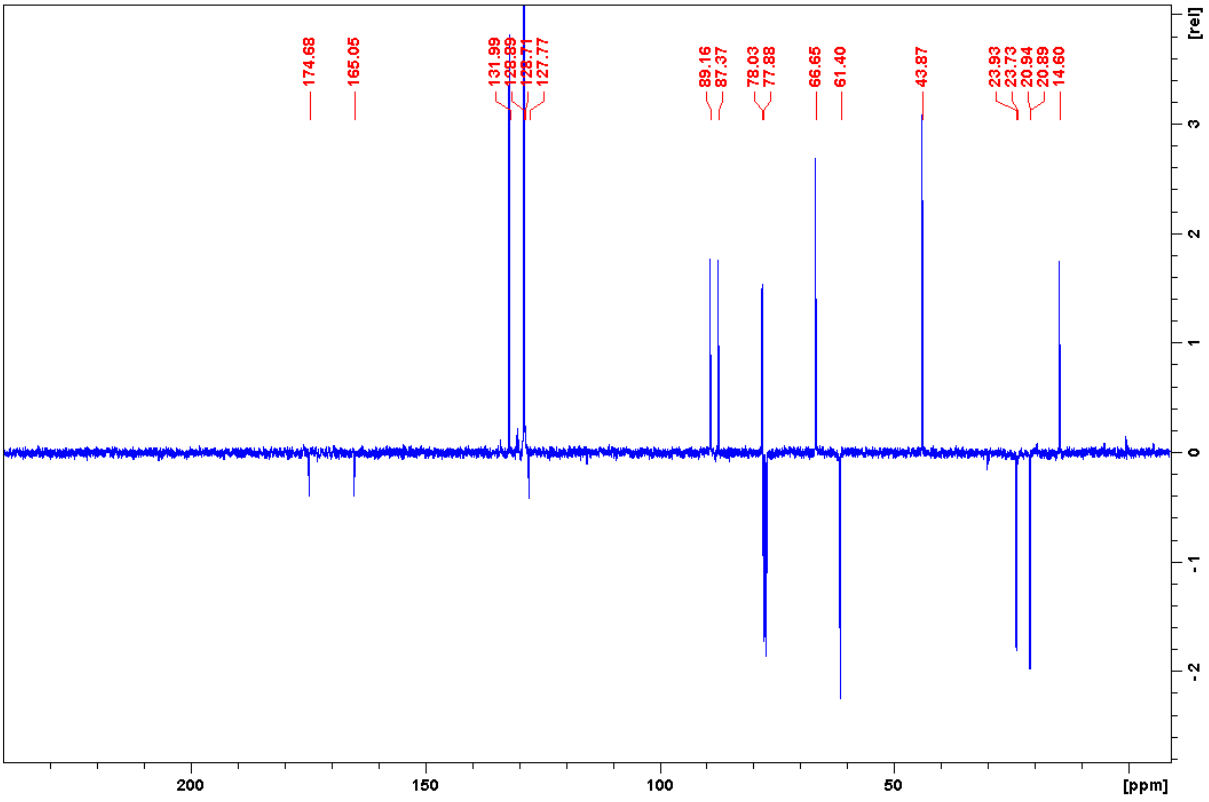
174.68
165.05
131.99
128.89
128.71
127.77
89.16
87.37
78.03
77.88
66.65
61.40
43.87
23.93
23.73
20.94
20.89
14.60
200
150
100
50
[ppm]
[rel]

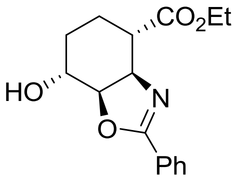

(±)-21

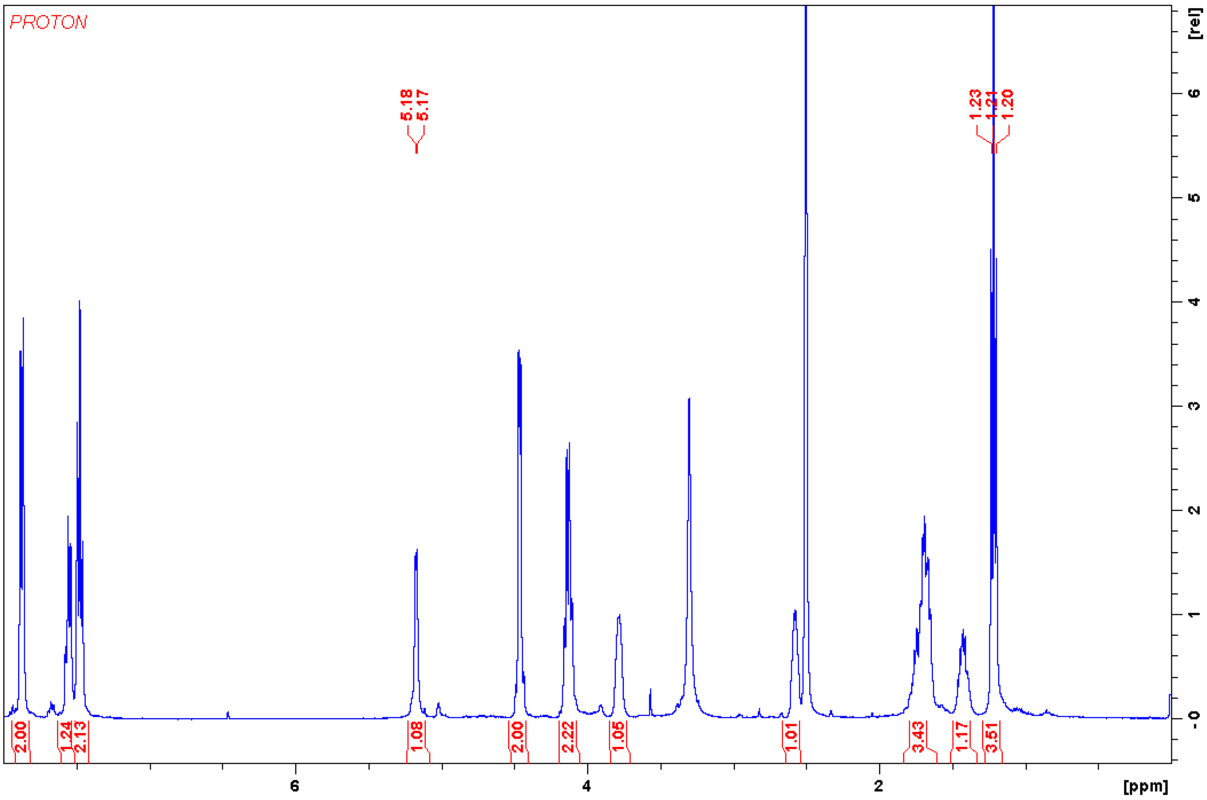
PROTON
5.18
5.17
1.23
1.21
1.20
2.00
1.24
2.13
1.08
2.00
2.22
1.05
1.01
3.43
1.17
3.51
6
4
2
[ppm]
[rel]

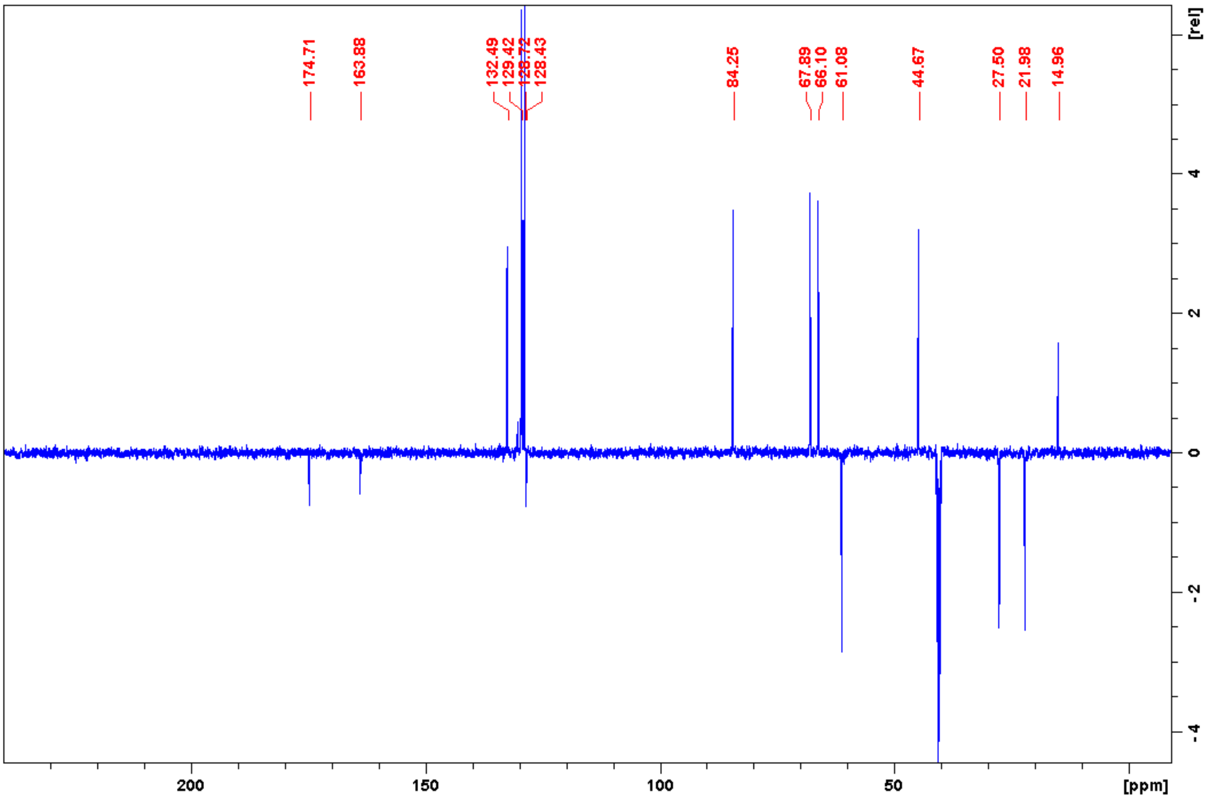
174.71
163.88
132.49
129.42
128.72
128.43
84.25
67.89
66.10
61.08
44.67
27.50
21.98
14.96
200
150
100
50
[ppm]
[rel]

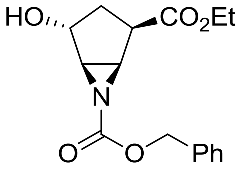
HO
CO2Et
N
O
O
Ph

(±)-7

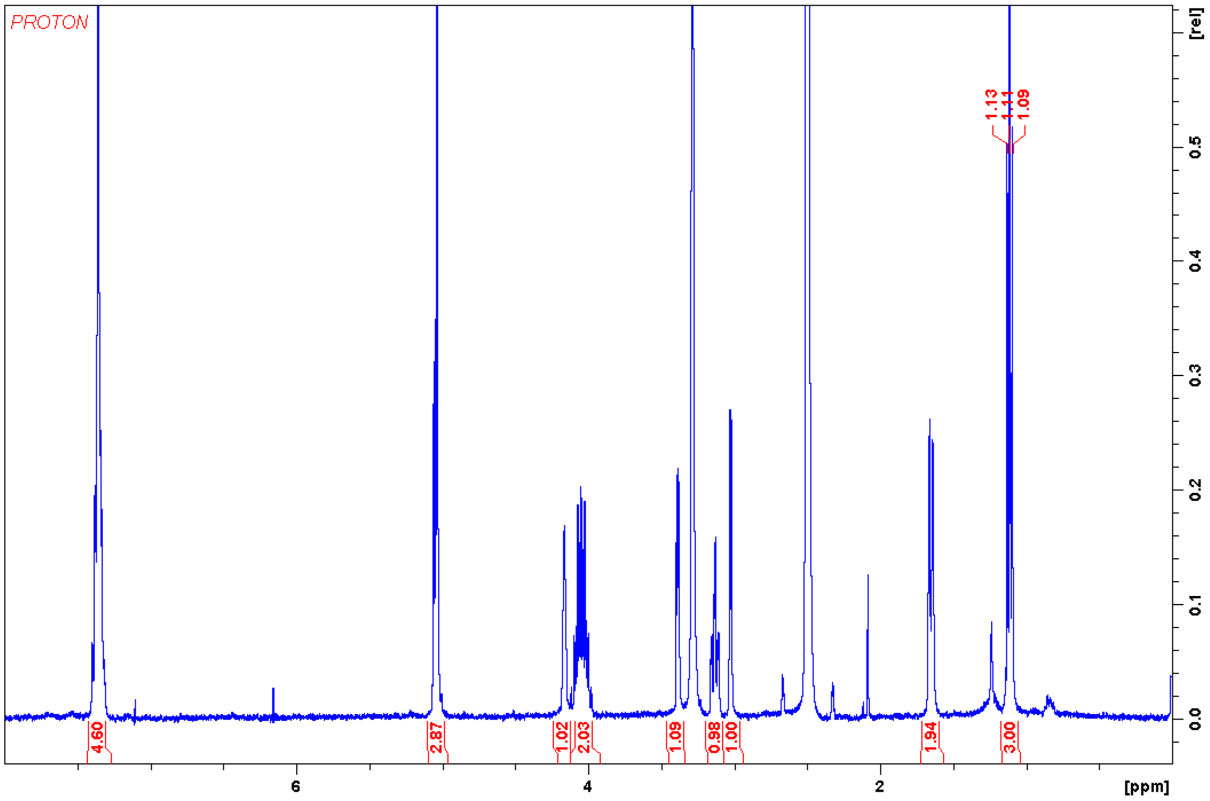
PROTON
1.13
1.11
1.09
4.60
2.87
1.02
2.03
1.09
0.98
1.00
1.94
3.00
6
4
2
[ppm]
[rel]
0.0
0.1
0.2
0.3
0.4
0.5

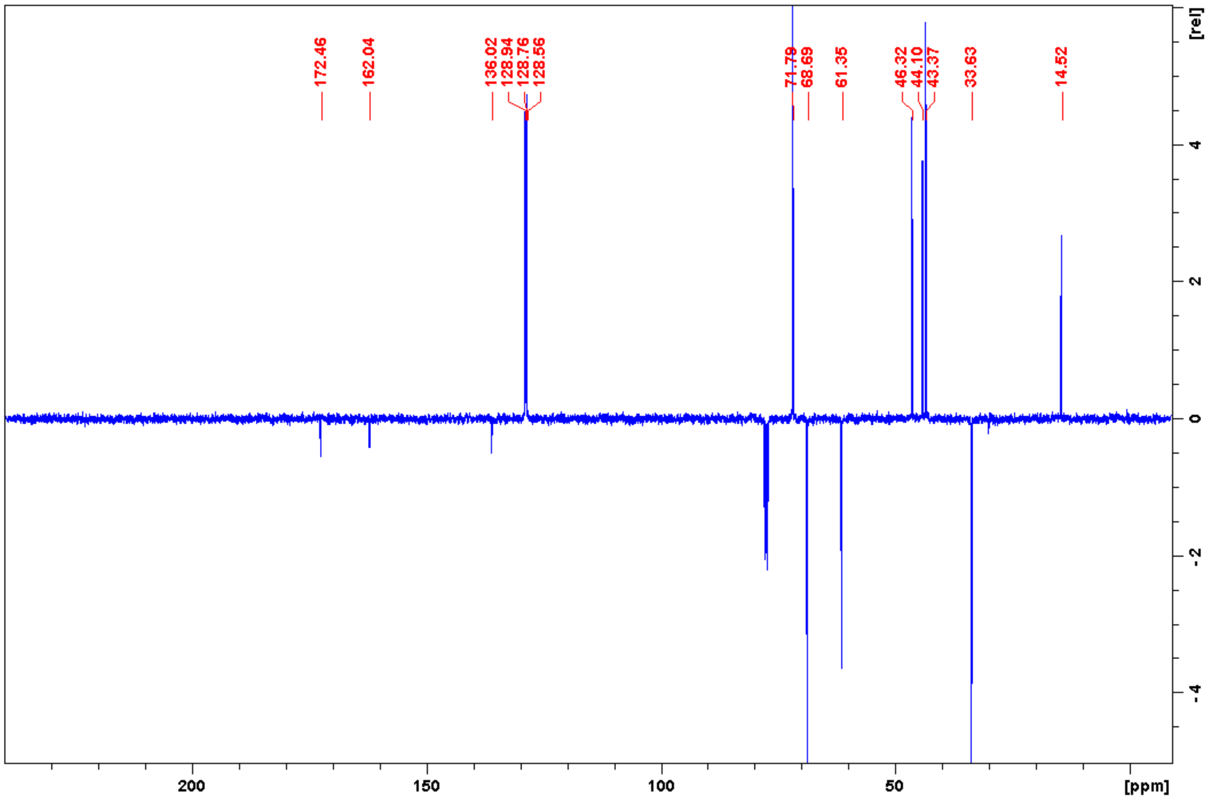
172.46
162.04
136.02
128.94
128.76
128.56
71.79
68.69
61.35
46.32
44.10
43.37
33.63
14.52
200
150
100
50
[ppm]
[rel]
4
2
0
-2
-4

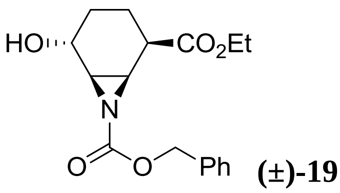
HO
CO2Et
N
O
O
Ph

(±)-19

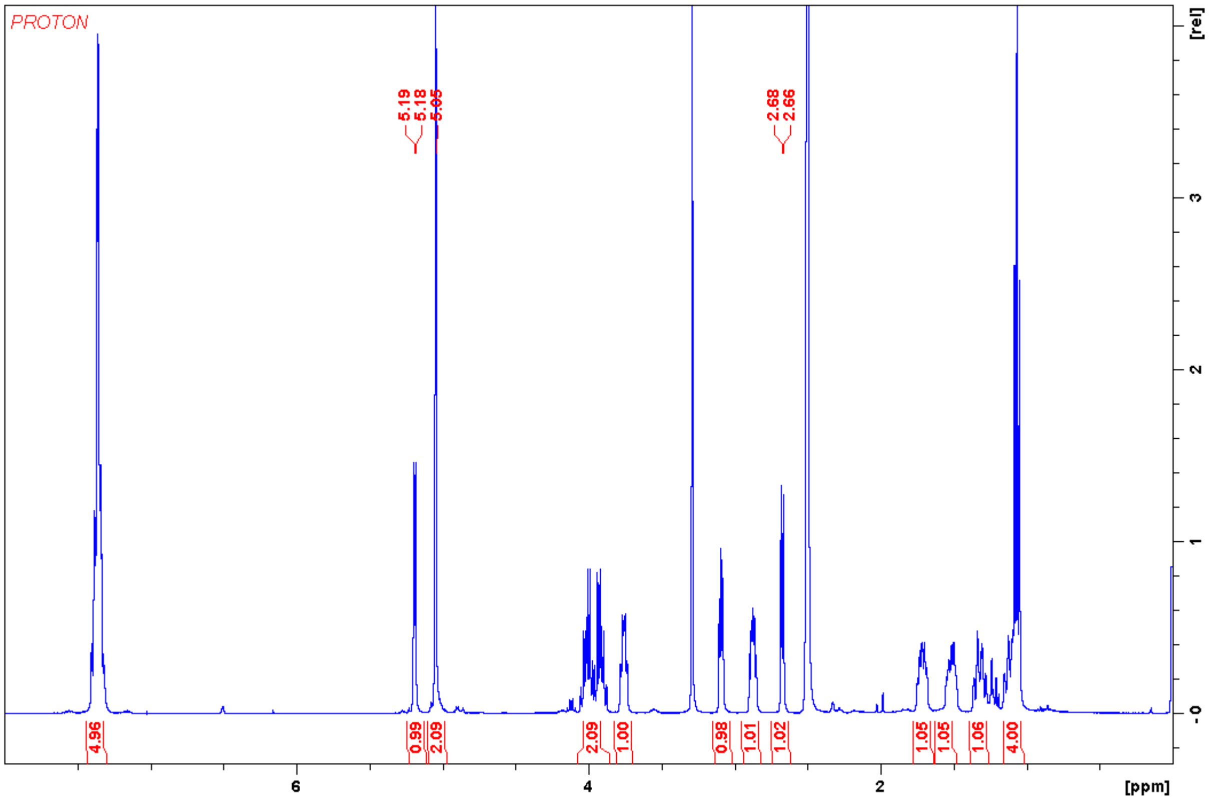
PROTON
5.19
5.18
5.05
2.68
2.66
4.96
0.99
2.09
2.09
1.00
0.98
1.01
1.02
1.05
1.05
1.06
4.00
6
4
2
[ppm]
[rel]

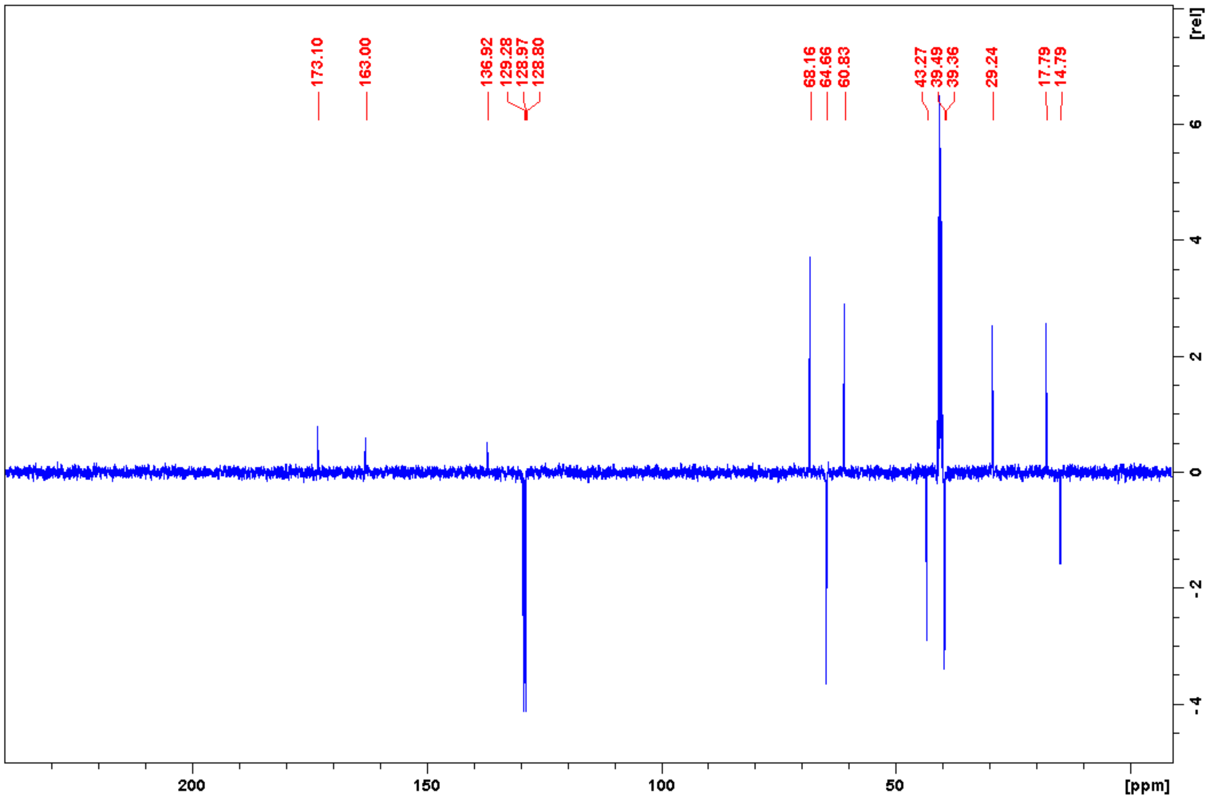
173.10
163.00
136.92
129.28
128.97
128.80
68.16
64.66
60.83
43.27
39.49
39.36
29.24
17.79
14.79
200
150
100
50
[ppm]
[rel]
